# Analysis of RNA base modification and structural rearrangement by single-molecule real-time detection of reverse transcription

**DOI:** 10.1186/1477-3155-11-8

**Published:** 2013-04-03

**Authors:** Igor D Vilfan, Yu-Chih Tsai, Tyson A Clark, Jeffrey Wegener, Qing Dai, Chengqi Yi, Tao Pan, Stephen W Turner, Jonas Korlach

**Affiliations:** 1 Pacific Biosciences, Menlo Park, CA, 94025, USA; 2 Department of Biochemistry & Molecular Biology, University of Chicago, Chicago, IL, 60637, USA

## Abstract

**Background:**

Zero-mode waveguides (ZMWs) are photonic nanostructures that create highly confined optical observation volumes, thereby allowing single-molecule-resolved biophysical studies at relatively high concentrations of fluorescent molecules. This principle has been successfully applied in single-molecule, real-time (SMRT®) DNA sequencing for the detection of DNA sequences and DNA base modifications. In contrast, RNA sequencing methods cannot provide sequence and RNA base modifications concurrently as they rely on complementary DNA (cDNA) synthesis by reverse transcription followed by sequencing of cDNA. Thus, information on RNA modifications is lost during the process of cDNA synthesis.

**Results:**

Here we describe an application of SMRT technology to follow the activity of reverse transcriptase enzymes synthesizing cDNA on thousands of single RNA templates simultaneously in real time with single nucleotide turnover resolution using arrays of ZMWs. This method thereby obtains information from the RNA template directly. The analysis of the kinetics of the reverse transcriptase can be used to identify RNA base modifications, shown by example for N6-methyladenine (m^6^A) in oligonucleotides and in a specific mRNA extracted from total cellular mRNA. Furthermore, the real-time reverse transcriptase dynamics informs about RNA secondary structure and its rearrangements, as demonstrated on a ribosomal RNA and an mRNA template.

**Conclusions:**

Our results highlight the feasibility of studying RNA modifications and RNA structural rearrangements in ZMWs in real time. In addition, they suggest that technology can be developed for direct RNA sequencing provided that the reverse transcriptase is optimized to resolve homonucleotide stretches in RNA.

## Background

The function and dynamic regulation of all forms of RNA are critically dependent on their sequence, structure, and post-transcriptional modifications [1]. Many different methods have been developed to interrogate RNA, but each tends to be sensitive to only one of these characteristics. For example, standard sequencing of RNA requires conversion into cDNA which causes the loss of information about structure and certain base modifications. In addition, these bulk methods average over a large ensemble of RNA molecules, thereby leaving potential complexities present in heterogeneous samples unresolved. With the advent of single-molecule techniques, it has become possible to sequence individual molecules of RNA directly by adaptation of a short-read DNA sequencing method to reverse transcription [2], however this method is still insensitive to RNA modifications and structure.

**Figure 1:**
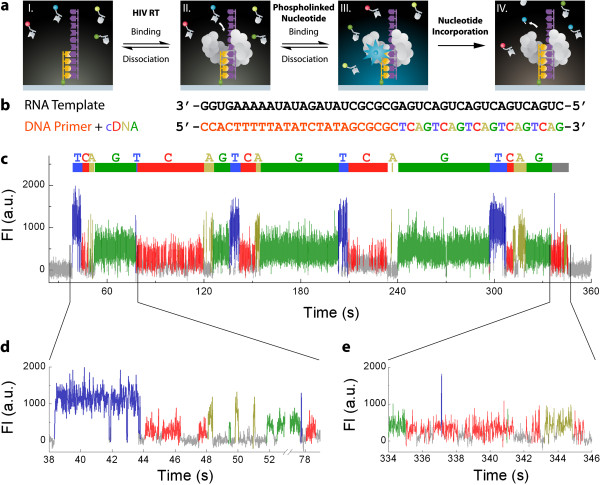
***Single-molecule, real-time (SMRT) reverse transcription in zero-mode waveguides (ZMWs).*** (**a**) Schematic of the SMRT reverse transcription process. The RNA template (purple) is hybridized to a biotinylated DNA primer (orange) and immobilized at the ZMW bottom (I). Reverse transcriptase (RT, gray) binds to the immobilized hybrid (II). Upon initiation of reverse transcription, a correctly base-paired phospholinked nucleotide binds in the enzyme’s active site (III). The bound nucleotide can either dissociate from the complex (reverse reaction), or become incorporated into the growing DNA chain, accompanied by the release of the labeled pyrophosphate (IV). HIV RT then translocates to the next position and the reaction cycle (II) through (IV) repeats. (**b**) Hybrid for demonstrating SMRT reverse transcription, showing the RNA template (purple), DNA primer (orange), and resulting DNA (base-specific color-coding). (**c**) Example trace of SMRT reverse transcription. Fluorescence pulses are color-coded as in (**b**). Each block of pulses belonging to the same nucleotide is indicated above the trace. The gray block at the end of the trace likely corresponds to non-templated cognate sampling. (**d-e**) Magnified views of the beginning and end of the trace, respectively.

We set out to adapt a technique originally developed for single-molecule, real-time (SMRT) DNA sequencing towards utilizing an HIV reverse transcriptase (HIV RT) for concurrent determination of RNA sequence, base modifications, and structure. Analogous to SMRT DNA sequencing [3], the RT activity is visualized by phospholinked deoxyribonucleotides that carry the fluorophore attached to the terminal phosphate moiety (Figure 1a). This allows for a continuous detection of cDNA synthesis in real time, as the fluorescent label is removed by the RT activity during nucleotide incorporation. Single RNA molecules are immobilized via a biotinylated primer inside zero-mode waveguides (ZMWs), and are independently and asynchronously interrogated on arrays containing thousands of ZMWs. The wild type HIV RT used in this study does not enable the resolution of the homonucleotide stretches. Nevertheless, by analyzing the kinetics of HIV RT we demonstrate the ability to detect base modifications in the RNA template, as well as obtain information on RNA secondary structure and its rearrangements. We also find that by following single-molecule reverse transcription in real time, it is possible to obtain sequence information from the RNA template directly.

## Results and discussion

### Single-molecule real-time reverse transcription of RNA

A development of direct SMRT RNA sequencing requires a processive RNA-dependent polymerase that can utilize phospholinked nucleotides. Reverse transcriptases represent one such class of enzymes which initiate polymerization on a DNA-primed RNA template and synthesize a complementary DNA strand. We evaluated an in-house HIV, and commercially available AMV as well as MMLV RTs for their ability to incorporate phospholinked nucleotides during first strand synthesis. A FAM-labeled DNA primer (FAM-P5) was hybridized to a synthetic RNA template (Additional file 1: Table S1) followed by the addition of RT with either native nucleotides or phospholinked nucleotides (Additional file 1: Figure S1a). The reactions were stopped by the addition of excess EDTA; the samples were denatured and run on a denaturing polyacrylamide gel (Additional file 1: Figure S1b). The fractions of templates utilized by the reverse transcriptases were determined by comparing the intensities of the band corresponding to the FAM-labeled DNA primer in the negative control and the reverse transcription reaction (Additional file 1: Figure S1b, c). In addition, we determined the fraction of the templates that yielded full-length first strand by comparing the intensity of the full length product in the reverse transcription to the intensity of FAM-labeled DNA primer in the negative control. A comparison of the data reveals that only HIV RT demonstrates an activity in the presence of phospholinked nucleotides that results in a detectable full-length product. Moreover, HIV RT shows comparable full-length activities in the presence of native and phospholinked nucleotides, with ~80% activity relative to the native dNTPs (Additional file 1: Figure 1b, c).

To demonstrate real-time detection of single-molecule reverse transcription in ZMWs, we employed HIV RT in conjunction with the synthetic RNA template containing a sequence signature of five consecutive repeats of 5’-CUGA-3’ at its 5’-end (Figure 1b). Upon addition of HIV RT to primed RNA templates immobilized in ZMWs, reverse transcription was detected by the appearance of fluorescent pulses (Figure 1c, Additional file 1: Figure S2). The color of the fluorescent pulses indicated an incorporation sequence comprising five repeated cycles of ‘TCAG’, matching the expected order of nucleotide incorporations on this RNA template. However, unlike SMRT DNA sequencing in which we introduced compensatory mutations to the DNA polymerase to restore the kinetics of phospholinked nucleotides to a single fluorescent pulse per each incorporation event [3], here multiple fluorescence pulses of the same nucleotide type are observed for each incorporation cycle (Figure 1d). Such blocks of like pulses are indicative of repeated binding and dissociation of correctly base-paired phospholinked nucleotides in the enzyme’s active site before eventual incorporation into the growing DNA chain. Pre-steady state kinetic measurements using bulk methods confirmed this interpretation, showing higher nucleotide dissociation rates compared to incorporation rates (Additional file 1: Figure S3, Additional file 1: Table S3). The succession of blocks can be used to determine the sequence of this RNA template, indicated by the color-coded delineation above the example trace in Figure 1c. While the groups of fluorescent signals for each incorporation cycle increase the confidence of RNA base calling, they currently preclude the ability to accurately sequence RNAs containing homonucleotide stretches. The incorporation time for a single nucleotide varied as a function of nucleotide type, with average block durations for A, C, G, and T phospholinked nucleotides 12 s, 20 s, 16 s, and 6 s, respectively (Additional file 1: Figure S4).

Upon completion of reverse transcription of the RNA template, fluorescence pulse activity ceased for the majority of SMRT reverse transcription reactions (data not shown). For some RNA molecules, more random phospholinked nucleotide binding events continued beyond this expected termination point (Figure 1e, Additional file 1: Figure S2), potentially reflecting non-templated extension at the end of first DNA strand synthesis previously reported for HIV RT [4].

### Detecting N6-methyladenine (m^6^A) modification in RNA

N^6^-methyladenine (m^6^A) is the most abundant modification in mRNAs of multicellular organisms and is also present in viral RNAs that replicate in the nucleus [5-7]. The proposed function of m^6^A in mRNA includes modulations of mRNA stability, splicing, and export from the nucleus. m^6^A modification has also been shown to confer innate immune tolerance of transfected exogenous RNA [8]. siRNA knockdown of the catalytic subunit of m^6^A methyltransferase led to cell apoptosis [6]. Despite the biological significance of m^6^A modification in mRNA, to date only one modification site is known in a specific mRNA due to the extremely laborious biochemical assays for mapping m^6^A sites in mRNA [6]. Current RNA sequencing methods cannot map m^6^A because Watson-Crick base pairing is not affected by the methyl group during cDNA conversion, thus causing the information about the base modification to be erased in that step. Recently, a combination of immunoprecipitation and next-generation sequencing enabled a localization of m^6^A in mRNAs within ~100 nucleotide long transcriptome regions [7,9,10].

**Figure 2:**
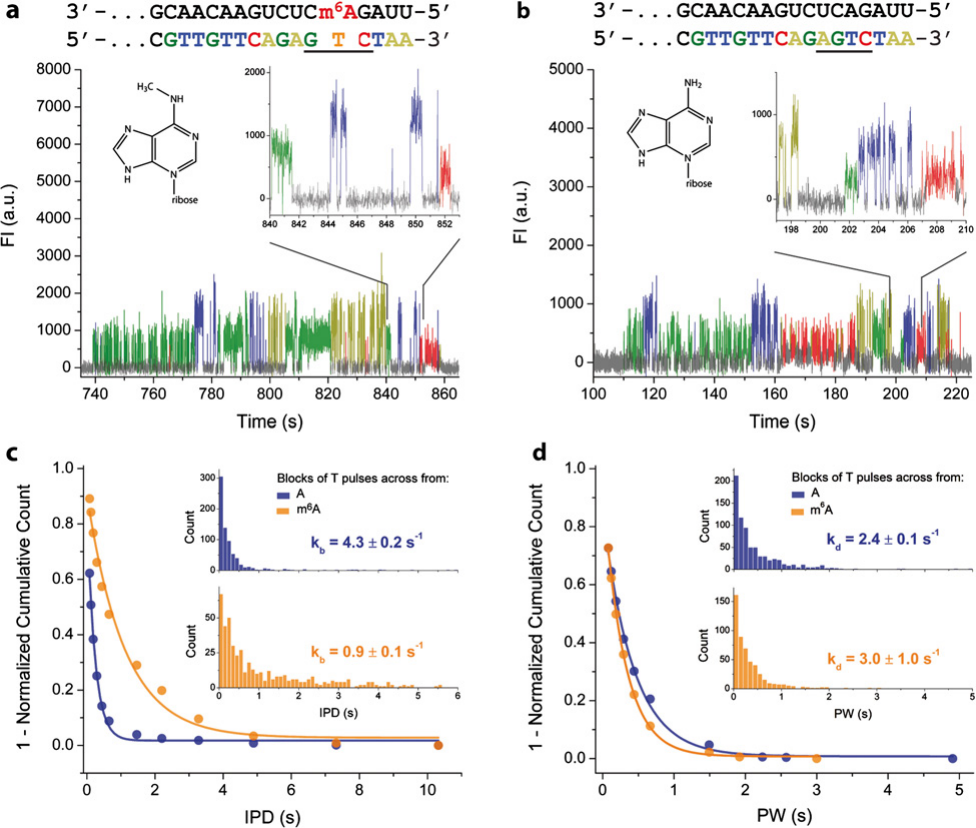
***Detection of modified RNA bases through the kinetics of SMRT reverse transcription.*** (**a**-**b**) Sequences and example traces of a SMRT reverse transcription trace on m^6^A-containing and unmodified A-containing control template, respectively. Enlarged trace regions show reverse transcription signal for the underlined sequences. (**c**-**d**) Distributions of (**c**) interpulse durations (IPDs) and (**d**) pulse widths (PWs) for phospholinked T incorporations across m^6^A (orange) and A (blue). The insets in (**c**) and (**d**) show histograms of the experimental counts used to determine the cumulative distributions. Phospholinked T binding (*k*_*b*_) and dissociation (*k*_*d*_) rates were obtained from single exponential fits to the distributions.

We have previously shown that DNA base modifications can be detected by their effect on the kinetics of DNA polymerase during SMRT DNA sequencing [11]. To test the potential of detecting RNA base modifications using the SMRT reverse transcription assay, we used the above-described assay to compare a synthetic RNA template containing m^6^A and an unmodified control template of the same sequence (Figure 2). In the representative SMRT reverse transcription time traces from synthetic m^6^A-containing and control RNA templates, the frequency of fluorescence pulses at the m^6^A position was decreased compared to the native A (Figure 2a, b), indicating that phospholinked nucleotide binding is affected by m^6^A in the RNA template. A comparison of the distribution of interpulse durations (IPDs) showed that m^6^A decreased the binding rate of the complementary phospholinked T nucleotide in the active site of the enzyme by ~5-fold (Figure 2c). Dissociation rates were not notably affected by the presence of m^6^A in the RNA template, as similar pulse width distributions were observed for both the m^6^A-containing and control template (Figure 2d).

**Figure 3:**
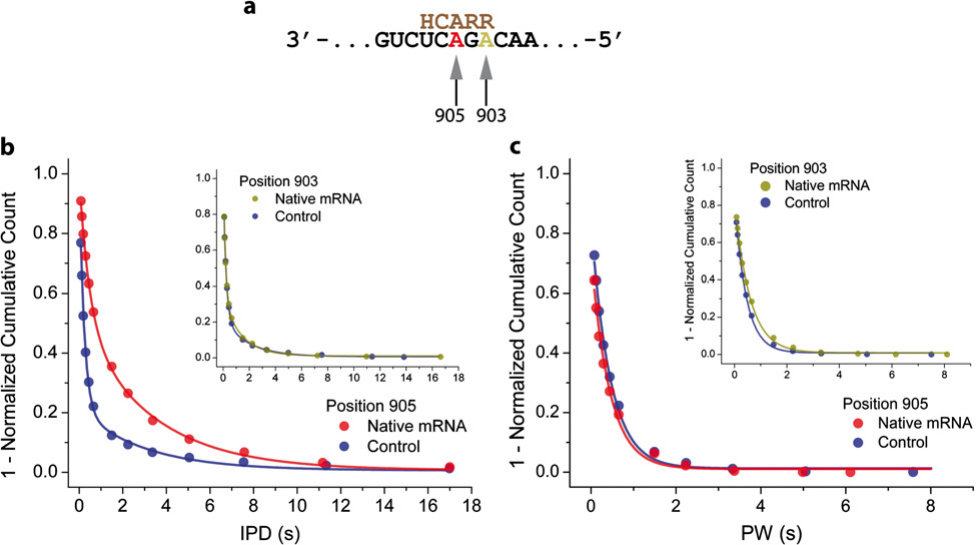
***Detection of m***^***6***^***A in native mRNA.*** (**a**) The section of the native mRNA containing the m^6^A modification sequence is shown in black with the potential site of methylation at position 905 shown in red and unmodified A at position 903 shown in dark yellow. The methyltransferase recognition consensus sequence is indicated in brown where R represents a purine, and H is a non-guanine base. (**b**) The main plot shows IPD distribution for phospholinked T incorporations across position 905 in native mRNA (red) and the *in vitro* transcribed control (blue), the Inset shows IPD distributions obtained at sequence position 903 in native mRNA (dark yellow) and the *in vitro* transcribed control (blue). (**c**) Pulse width (PW) distributions for phospholinked T incorporations across from the consensus site for m^6^A modification (position 905; data obtained with native mRNA and *in vitro* transcribed control shown in red and blue, respectively). Inset: PW distribution for phospholinked T incorporations at the unmodified adenosine control at position 903 (data obtained with native mRNA and *in vitro* transcribed control shown in dark yellow and blue, respectively).

Next, we tested the ability to detect m^6^A in RNA isolated from a biological sample. A plasmid containing a known m^6^A motif from bovine prolactin mRNA in the 3’ UTR of a GFP gene was transfected in breast cancer cells. Total mRNA was isolated after 24 h and subjected to the SMRT reverse transcription assay (Figure 3). The distribution of IPDs at the site of potential m^6^A modification (position 905) obtained on native mRNA revealed a slower binding kinetics compared to the *in vitro* transcribed control, while the dissociation kinetics remained unaffected (Figure 3b, and Figure 3c, respectively). In contrast, the kinetics of phospholinked T binding at a neighboring site in the RNA template sequence (position 903) was indistinguishable in native mRNA and the control template (insets in Figure 3b, and Figure 3c). The observed kinetic signal is thus consistent with an m^6^A modification at position 905 in the native mRNA.

### Analysis of RNA secondary structure

RNA secondary structure is an important modifier of biological functions and activity such as transcription, post-transcriptional modifications, and translation. Furthermore, RNA structure rearrangement progressing in a 3’ to 5’ direction may play important roles in biological function. For instance, reverse transcription of the retroviral RNA genome or cellular retro-transposon RNA consecutively removes RNA segments from RNA structural formation, so that the remaining, not yet reverse transcribed RNA can refold into other structures as reverse transcription progresses [12,13]. Similarly, mRNA degradation by 3’ to 5’ exonuclease or 3’ to 5’ exosome results in consecutive removal of RNA segments from the 3’ end, so that the remaining, not yet degraded RNA can fold into distinct structures as the exosome moves along the mRNA [14,15].

**Figure 4:**
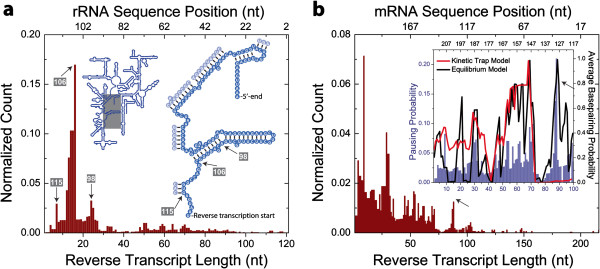
***Detection of RNA structure rearrangement during SMRT reverse transcription.*** (**a**) Histogram of reverse transcript lengths on *E. coli* 16S rRNA. The top axis shows 16S rRNA sequence position as defined by EcoCyc [17]. The left inset shows a schematic of 16S rRNA secondary structure. A section of 16S rRNA (gray rectangle) is expanded in the right inset, showing the start of SMRT reverse transcription and the 5’-end of 16S rRNA. Reverse transcribed bases are shown as solid blue circles and bases involved in secondary structures in transparent blue circles. Peaks in the histogram and their corresponding 16S rRNA sequence positions are indicated by arrows. (**b**) Histogram of reverse transcript lengths on human ribosomal protein S17 *mRNA*. Inset: Pausing probabilities along the mRNA template (blue bars), compared to calculated base-pairing probabilities of this mRNA template using models that do not allow (red) or do allow (black) for refolding of the RNA template remaining at each position in the DNA synthesis (Kinetic Trap Model and Equilibrium Model, respectively). A pause along mRNA template during SMRT reverse transcription was defined as stalling of reverse transcription for longer than 5 min (Methods).

It has previously been shown that reverse transcription kinetics is sensitive to RNA secondary structure [16]. To test whether our method is sensitive to structural features in the RNA template, we first interrogated a structurally well-defined RNA – *E. coli* 16S ribosomal RNA – by SMRT reverse transcription (Figure 4a). The primer extension reaction was initiated at sequence position 121 (right inset in Figure 4a). The presence of the homonucleotide stretches in section of *16S rRNA* sequenced prevented determination of the exact length of reverse transcripts when the last aligned block corresponded to a homonucleotide stretch. For this reason, we have merged each homonucleotide stretch of *16S rRNA* sequence into one single *homonucleotide block* resulting in a *collapsed 16S rRNA* sequence (Additional file 1: Table S4) that was then used in the alignment of the data. The observed number of reads ending within each homonucleotide block was equally distributed between the composite bases by dividing the number of the observed reads within a homonucleotide block by the number of the constituting bases. The histogram of the aligned reverse transcript lengths showed a peak at sequence position 106 which represents the start of an extended RNA stem (Figure 4a). Two minor peaks, at sequence positions 98 and 115, indicated that the method is also sensitive to detect more subtle RNA structures, in this case an RNA bulge or a short stem, respectively. The product lengths exceeding 100 nts were not readily detected due to the stable secondary structure of the *16S rRNA*. The secondary structure increases the probability of long pauses or termination events [16]. Consequently, each base-paired template position decreases the probability of HIV RT to extend the nascent strand and to translocate. A succession of basepaired positions will thus gradually decrease the number of longer product lengths until the position beyond which undetectable number of HIV RTs will progress.

Unlike ribosomal RNA, the majority of RNAs lack a single stable RNA fold. Instead, at equilibrium, RNA molecules with the same sequence adopt folds with different basepairing combinations. This means that the same nucleotide in an RNA sequence can be basepaired in one fraction of these folds while it is not basepaired in the remainder. As shown previously by Kim et al. [18], the basepairing state of the base will determine the average rate of nucleotide incorporation during reverse transcription at that position. In case of a basepaired nucleotide, the incorporation of the complementary nucleotide in the nascent DNA strand will be more likely to induce HIV RT pausing or termination [16,19]. Consequently, the probability to observe a pausing or termination event at a particular sequence position of RNA will depend on the fraction of molecules where this sequence position is in a basepaired state.

In order to test whether SMRT reverse transcription would provide structural information on RNAs lacking a single stable fold, we transcribed mRNA of human 40S ribosomal protein S17 *in vitro* (Additional file 1: Table S1) and performed the SMRT reverse transcription assay. The resulting histogram of aligned reverse transcript lengths displayed a pattern with several peaks (Figure 4b). To test whether the observed peaks correlate with RNA sequence positions prone to form base-paired secondary structures, we compared the normalized experimental pausing data to calculated base-pairing probabilities using Kinetic trap model (Inset in Figure 4b; blue bars, and red line, respectively, Methods). We observed good correlation of the data with the predicted degree of secondary structure for reverse transcripts shorter than 75 nucleotides in length, as judged by Spearman correlation analysis (Additional file 1: Figure S5). However, this analysis failed to predict the presence of a strong pause site observed experimentally at position 86 (black arrows in Figure 4b). As reverse transcription converts the RNA template into a DNA/RNA hybrid, the initial secondary structures in the RNA template are progressively removed. We therefore hypothesized that during the course of DNA synthesis, the remaining RNA template may refold, rearranging RNA secondary structure and consequently altering basepairing probabilities [18]. We thus calculated basepairing probabilities using a model where the structure of the yet untranscribed RNA template was allowed to re-equilibrate prior to nucleotide incorporation (Equilibrium model in Methods). The basepairing probabilities calculated using this model (Inset in Figure 4b, black line, Additional file 1: Figure S6) captured the later pausing behavior observed in SMRT reverse transcription assay, demonstrating that SMRT reverse transcription not only allows the detection of relatively static, stable RNA structures, but may also allow the real-time interrogation of structural rearrangements in RNA.

## Conclusion

In contrast to SMRT DNA sequencing, the SMRT reverse transcription assay utilizing HIV-RT produces multiple binding events for each nucleotide incorporation and relatively short read-lengths. This is not surprising since this enzyme has not yet been optimized for incorporation of phospholinked nucleotides, and is reminiscent to the performance of the SMRT DNA sequencing at the starting point. We are in the process of engineering RTs to obtain a mode where each nucleotide binding event results in a successful incorporation, through changes in the enzyme analogous to our previous DNA polymerase engineering [3]. In addition, the observed read-lengths (Figure 4b) are shorter than previously reported in vitro processivities of HIV RT [20]. This is due to the use of nucleotide analogues in place of the native nucleotides. Our bulk studies suggest that the ability of HIV RT to progress through RNA template is hampered by the bulky nature of the fluorescence probes attached to the nucleotide analogues (data not shown). Read-lengths can be improved by increasing the RT’s processivity in the presence of nucleotide analogues through mutations, or by employing viral RNA-dependent RNA polymerases (RdRPs) that exhibit extremely high processivities on RNA templates [21]. For example, we have evaluated a member of this class of enzymes, ϕ6 RdRP, and found that it can utilize phospholinked nucleotides (Additional file 1: Figure S7).

In summary, we have demonstrated the ability to observe reverse transcription using SMRT technology, allowing information about sequence content, base modifications, and structural rearrangement of RNA to be obtained simultaneously. We were able to detect m^6^A modifications in a specific mRNA from a mixture of total cellular mRNA, and to identify structural rearrangements in an mRNA. We anticipate that the application of our method may enable the identification of the location of many modified bases in mRNA and provide detailed information about the nature and the dynamic RNA refolding in retroviral/retro-transposon reverse transcription and in 3’-5’ exosome degradation of mRNA.

## Methods

### Reverse transcriptase preparation

Methods for expression and purification of HIV reverse transcriptase (HIV RT) were adapted from previously published procedures [22,23]. *E. coli* expression vectors for HIV RT p66 and His-tagged p51 subunits were custom synthesized by DNA 2.0 (Menlo Park, CA). BL21 *E. coli* were transformed with each expression vector and cultured overnight. After 100-fold dilution into fresh LB media, the cultures were grown further at 37°C. The incubator temperature was reduced to 18°C when the *E. coli* density reached 0.9 OD_600_ followed by the addition of 1 mM IPTG for induction. The cultures were allowed to grow overnight before harvesting.

Cell pellets from the p66 and p51 cultures were combined in 2:1 weight ratio and re-suspended in lysis buffer (30 mM HEPES pH 7.5, 60 mM NaCl, 2 mM EDTA, 1 mM DTT, and 1mM PMSF). The cell suspension was passed through a microfluidizer device 3 times at 80 psi (Microfluidics, Newton, MA) to lyse the cells. HIV RT was released from bacterial DNA by adding 0.5 M NaCl to the lysate and stirring for 30 min at 4°C. PEI was then slowly added to the stirring lysate to 0.3% final concentration and stirred for another 30min. Precipitated bacterial DNA was removed by centrifugation. Ammonium sulfate was added at 0.37 g/ml to the cleared lysate and precipitated proteins were collected by centrifugation. Protein pellets were re-suspended with 30 mM HEPES pH 7.5 and buffer content adjusted to Buffer A (30 mM HEPES pH 7.5, 500 mM NaCl, and 10 mM imidazole). The HIV-RT was then purified using a HisTrap FF crude column (GE Healthcare, USA) and a 10 mM to 500 mM imidazole linear gradient. Collected fractions containing HIV-RT heterodimer were pooled and concentrated using a 30k MWCO Vivaspin 4 device (Sartorius Stedim Biotech, France). A Superdex 200 column was used for the final purification step. Purified HIV RT was stored at −20°C in a buffer containing 47.5% glycerol, 30 mM HEPES pH 7.5, 60 mM NaCl, 0.1 mM EDTA, and 1 mM DTT. The final concentration was determined by UV absorbance and extinction coefficients calculated from the amino acid sequences.

### Nucleic acid preparation

All nucleic acids used in the study are listed in Additional file 1: Tables S1 and Additional file 1: S2. All DNA primers as well as synthetic RNA and ssRNA templates were ordered from IDT and dissolved in 1x TE.

m^6^A and m^6^A control templates were synthesized as described previously [24]. DNA sequence for native mRNA was designed to contain the 3’-UTR sequence from bovine prolactin mRNA (NCBI Reference Sequence: NM_173953.2), followed by an adaptor (5’-GCGCGCTATAGATATAAAAAGTGG). This DNA sequence was cloned into the pEGFP-C1 plasmid (Clontech), between XhoI and BamHI sites. The plasmid was then transfected into MDA-MB-231 cells (~85-90% confluent) with Lipofectamine 2000 (Invitrogen), following the procedure suggested by the manufacturer. GFP level was monitored after 24 h of transfection to insure the expression of GFP protein, and thus the production of the designed native mRNA. polyA+ RNA was subsequently isolated with FastTrack 2.0 mRNA isolation kit (Invitogen). Control for the native mRNA was obtained through *in vitro* run-off transcription using T7 RNA polymerase and the template obtained with PCR of the plasmid used in the transfection.

Ribosomal RNA from *E. coli* was purchased from Applied Biosystems as solution of total RNA from *E. coli*. mRNA template was synthesized by *in vitro* run-off transcription using cDNA of 216 nt long 3’-end section of the human 40S ribosomal protein S17 gene and T7 promoter, as described previously [23].

### Nucleic acid hybridization

All complementary nucleic acids were hybridized in 10 mM Tris, pH 7.5, 75 mM KCl and incubated at 65°C for 5 min followed by gradual cooling to 25°C at 0.1°C s^-1^ and cooled down to 4°C before storing at −20°C. All hybridization pairs used in this study are listed in Additional file 1: Tables S1 and Additional file 1: S2.

### Activity of reverse transcriptases (RT) in the presence of phospholinked nucleotides

We have tested in-house HIV RT and commercially available AMV RT (New England Biolabs, MA), and MMLV RT (New England Biolabs, MA) for their ability to incorporate phospholinked nucleotides. To this purpose, we have hybridized synthetic RNA template and FAM-P5 DNA primer (Additional file 1: Table S1) as described above.

For each reverse transcriptase tested, reaction mixtures with native and phospholinked nucleotides were prepared separately. Each mixture was prepared in duplicates and contained 2 μM DNA-primed synthetic RNA template, 3 μM per nucleotide, 100 units/mL reverse transcriptase, and 1x RT buffer. In the case of HIV RT, the reaction buffer described below was used while recommended manufacturer reaction buffers were used for AMV and MMLV RTs. One of the mixture duplicates was supplemented with 45 mM EDTA for a negative control. All mixtures were incubated at 37°C for 1 h. The reverse transcriptions were then stopped with 45 mM EDTA, mixed with 2x volume of RNA Sample Loading Buffer (Sigma-Aldrich), and denatured for 10 min at 65°C. Denatured samples were run on a denaturing 15% PAGE, and analyzed on a Typhoon imager (GE Healthcare Life Sciences).

### Single nucleotide incorporation rates in reverse transcription

We have determined nucleotide binding rates *k*_1_, nucleotide dissociation rates *k*_−1_, and incorporation rates (*k*_2_) for phospholinked nucleotides using pre-steady state bulk kinetic assays (Additional file 1: Scheme S1). First, we determined binding and incorporation rates for each phospholinked nucleotide in a real-time stopped-flow experiment. DNA templates labeled with 6-carboxyfluorescein (FAM) at their 5’-ends were first hybridized to their corresponding DNA primer (Additional file 1: Table S2). Measurements were carried out in SMRT reverse transcription reaction buffer (50 mM Tris pH 8.3 at room temperature, 10 mM KCl, 0.05 mM CaCl_2_, 5 mM DDT, 0.2 units· μl^-1^ Superase•In™, 2 vol% FMP, 5 mM Trolox, 2 mM PCA, 1x PCD, 2.5 mM MgCl_2_). Changes in FAM fluorescence upon mixing of the nucleotide analog with RT-DNA template complex were recorded with a SF-2004 stopped-flow instrument (Kintek, Austin, TX). *k*_−1_ and *k*_2_ were determined by nonlinear regression either by the stopped-flow software or by the KaleidaGraph software using double-exponential and hyperbolic equations with data collected at various nucleotide concentrations.

To measure the dissociation rate of phospholinked nucleotides (*k*_−1_ in Additional file 1: S1), we hybridized the above DNA templates to dideoxynucleotide-terminated DNA primers (Additional file 1: S2). Ternary complexes of RT, phospholinked fluorescent nucleotides, and DNA template were prepared for a stopped-flow assay. The fluorescence signal was recorded as described above. Subsequently, *k*_−1_ was determined from the increase in fluorescence signal upon addition of unlabeled nucleotides.

### RNA replication with RNA-dependent RNA polymerase from Bacteriophage φ6 using phospholinked nucleotides

Stalling and replication was carried out as previously described by Dekker and coworkers [25]. Briefly, ssRNA template (Additional file 1: S1) was used as a template in the replication reaction. RNA-dependent RNA polymerase from Bacteriophage ϕ6 (ϕ6 RdRP) was first stalled on ssRNA in the presence of three ribonucleotides (rATP, rGTP, and rUTP), and reinitiated by the addition of native rCTP or phospholinked rCTP. Reinitiated replication was carried out at 32°C for 4 h. Reaction products were analyzed on a native PAGE.

### SMRT reverse transcription assay

Phospholinked dNTPs were generated as described previously [3,26]. ZMW nanostructures were fabricated and functionalized as previously described [18,27]. Single-molecule experiments were performed using arrays of 3,000 ZMWs monitored simultaneously, with excitation laser lines as described previously [3,28]. 5’-biotinylated DNA primer was hybridized to RNA template and incubated with streptavidin at 1:1 molar ratio in Immobilization Buffer (50 mM Tris pH 8.0, 10 mM KCl, 0.1 mM CaCl_2_, 5 mM DDT, 0.2 units·μl^-1^ Superase•In™ (Applied Biosystems, Carlsbad, CA)) at 37°C for 5 min and stored on ice. DNA primer-RNA template-streptavidin complex was immobilized in ZMWs by incubation on ZMW arrays for 5 min at room temperature in the Immobilization Buffer. Excess complex was removed by washing with Wash Buffer (50 mM Tris pH 8.3 at room temperature, 10 mM KCl, 0.05 mM CaCl_2_, 5 mM DDT, 0.2 units· μl^-1^ Superase•In™, 2.5 mM MgCl_2_). Next, 30 μl Reaction Buffer (50 mM Tris pH 8.3 at room temperature, 10 mM KCl, 0.05 mM CaCl_2_, 5 mM DDT, 0.2 units· μl^-1^ Superase•In™, 2 vol% FMP, 5 mM Trolox, 2 mM PCA, 1x PCD, 2.5 mM MgCl_2_, and 3 μM of each phospholinked nucleotide) was applied to the array and placed in the instrument. The recording of the fluorescent signals was started and reverse transcription on the chip was initiated by adding 10 μl of Enzyme Buffer (Reaction Buffer supplemented with 2 nM HIV RT) at room temperature. The movie acquisition was stopped after 15 min. In the case of experiments carried out at 37°C, the above Reaction Buffer was replaced by Reaction Buffer 2 (50 mM Tris pH 8.5 at room temperature, 10 mM KCl, 0.05 mM CaCl_2_, 5 mM DDT, 0.2 units· μl^-1^ Superase•In™, 2 vol% FMP, 5 mM TSQ, 5.8 mM PCA, 1x PCD, 60 μg· ml^-1^ BSA, 2.5 mM MgCl_2_, 3 μM per nucleotide analogue; ionic strength at 37°C approx. 35 mM). In addition, when the experiments were carried out at 37°C, the fluorescent signal was measured in 10 successive 5 min long movies with ZMW array alignment steps after each movie to correct for thermal drift.

### Data collection and analysis

Data were collected on a highly parallel confocal fluorescence detection instrument, as previously described [3,28]. Pulse calling, which uses a threshold algorithm of the dye-weighted intensities of fluorescence emissions, has been described previously [3]. The links to the raw data are listed in Additional file 1: Table S5.

### HIV RT pausing during SMRT reverse transcription

A pause along *mRNA* template during SMRT reverse transcription was defined as stalling of reverse transcription for longer than 5 min. The pausing probability for a given sequence position was then defined as a fraction of reverse transcription events pausing at that position relative to the total number of reverse transcripts containing this position.

### Basepairing probability on mRNA and SMRT reverse transcription pausing

We have applied two different models to calculate the basepairing probability for each sequence position of *mRNA* template when it is present in the active site of HIV RT. In the Kinetic Trap Model (*KTM*), an ensemble of full length *mRNA* folds was first generated using the program *mfold*[29] with the following conditions: folding temperature 37°C, suboptimality 33%, window parameter 1, and preventing basepairing of nucleotides hybridized to DNA primer (bolded nucleotides in Additional file 1: Table S1). Next, the resulting ensemble folds were used to calculate basepairing probabilities for every nucleotide of *mRNA* during the first nucleotide incorporation event:

$$P_{\mathrm{bp}}\left(1,j\right)=\frac{\sum_{k=1}^{n}e^{-\frac{\Delta G_{k}}{\mathrm{RT}}}}{\sum_{l=1}^{m}e^{-\frac{\Delta G_{l}}{\mathrm{RT}}}};j\geq 1$$

where *P*_*bp*_(1,*j*) is the basepairing probability for *j*-th nucleotide when the first nucleotide is bound in the RT active site, *n* is the number of *mfold* structures where *j*-th nucleotide is in a basepaired state, *m* is the number of all calculated *mfold* structures, *ΔG*_*k*_ is a free energy of folding a *k* structure, *ΔG*_*l*_ is a free energy of folding an *l* structure, *R* is the gas constant (8.314 J·(mol·K)^-1^), and *T* is reaction temperature (310.15 K). After the first nucleotide incorporation, RT translocates to the second nucleotide. The base pairs of the initial ensemble of *mRNA* folds are retained except for those involving the first base that has been reverse transcribed and is now forming part of the RNA/cDNA hybrid product. A set of basepairing probabilities *P*_*bp*_(2,*j*) was then determined using the readjusted ensemble of *mRNA* folds. This procedure was subsequently repeated for every *mRNA* sequence position. The calculated basepairing probabilities were corrected since the stability of a predicted base pair can depend on the basepairing state of its sequence neighbors [30]. We have thus calculated an average basepairing probability for each nucleotide when present in the active site of RT by averaging the above basepairing probabilities with a sliding window upstream of the nucleotide in the active site:

$$P_{\mathrm{bp}}^{'}\left(j,j\right)=\frac{\sum_{i=j}^{j+w}P_{\mathrm{bp}}\left(j,i\right)}{w}$$

where *P*_*bp*_*’*(*j*,*j*) is the average basepairing probability for *j*-th nucleotide when it is bound in the active site of HIV RT, and *w+1* is the width of the sliding window. We have calculated *P*_*bp*_*’*(*j*,*j*) values using *w* values of 0, 2, 4, 6, and 8 (Additional file 1: Figure S5a-e). The correlation between *P*_*bp*_*’*(*j*,*j*) values and experimentally measured pausing probability on *mRNA* was evaluated using the Spearman correlation coefficient (Additional file 1: Figure S4f). The coefficient increased from *w* = 0 to *w* = 4, where it plateaued above the critical value (0.38 at 99.9% confidence level and 73 degrees of freedom; green dashed line in Additional file 1: Figure S4f). Averaging beyond *w* = 4 did not improve the correlation coefficient notably.

In the Equilibrium Model (*EM*), the initial ensemble of mRNA folds and the basepairing probabilities were determined in the same way as described above for *KTM*. However, after the first nucleotide incorporation and RT translocation to the second base, the untranscribed *mRNA* now shortened by one nucleotide was allowed to re-equilibrate to a new ensemble of stable folds. A set of basepairing probabilities *P*_*bp*_(2,*j*) was then obtained using the re-equilibrated ensemble of *mRNA* folds. This re-equilibration procedure was subsequently repeated for every *mRNA* sequence position, and basepairing probabilities averaged as explained above for *KTM* using w = 4 (Additional file 1: Figure S5).

## Abbreviations

cDNA: complementary DNA; m6A: N6-methyladenine; SMRT: single-molecule real time; RT: reverse transcriptase; ZMWs: zero-mode waveguides; IPDs: interpulse durations; rRNA: ribosomal RNA; mRNA: messenger RNA; HIV: Human Immunodeficiency Virus; KTM: Kinetic Trap Model; EM: Equilibrium Model; RdRP: RNA-dependent RNA polymerase; ϕ6 RdRP: RNA-dependent RNA polymerase from Bacteriophage ϕ6; FAM: 6-carboxyfluorescein; AMV: Avian Myeloblastosis Virus; MMLV: Moloney Murine Leukemia Virus; GFP: Green Fluorescent Protein

## Competing interests

IDV, YT, TAC, SWT & JK are full-time employees at Pacific Biosciences, a company commercializing single-molecule, real-time nucleic acid sequencing technologies.

## Authors’ contributions

IDV performed the SMRT reverse transcription experiments, analysed the data, tested different RTs, and ϕ6 RdRP. YCT prepared and characterized HIV RT enzymes. TAC carried out bulk tests on HIV RT in the presence of fluorescently labelled nucleotide analogues. JW synthesized fluorescently labelled nucleotide analogues. QD synthesized the RNA templates with m^6^A. CY expressed the native mRNA template in vivo. IDV, TP, SWT and JK wrote the paper. All the authors read and approved the final manuscript.

## Supplementary Material

Additional file 1: Scheme S1 Reaction mechanism of reverse transcriptase. **Figure S1.** Activity of HIV, AMV and MMLV reverse transcriptases (RTs) in the presence of phospholinked nucleotides (PL-Ns). **Figure S2** (**a**)-(**j**)**.** Examples of SMRT reverse transcription traces obtained with synthetic RNA template. **Figure S3.** Determination of binding, dissociation and incorporation rates for HIV RT using phospholinked nucleotides. **Figure S4.** Cumulative distributions of block widths during SMRT reverse transcription for incorporations of A (yellow), C (red), G (green), and T (blue) phospholinked nucleotides by HIV RT in ZMWs. **Figure S5.** Basepairing probabilities on *mRNA* template calculated using the Kinetic Trap Model (KTM). **Figure S6.** Basepairing probabilities on *mRNA* calculated using the Equilibrium Model (*EM*). **Figure S7.** Activity of RNA-dependent RNA polymerase from bacteriophage ϕ6 (ϕ6 RdRP) in the presence of a phospholinked rCTP. **Table S1.** RNA templates and DNA primers used in SMRT reverse transcription. **Table S2.** Sequences of DNA templates and corresponding DNA primers used in the bulk measurements of HIV RT transcription kinetics (Additional file 1: Figure S1). **Table S3.** Bulk kinetics data obtained with stopped-flow experiments (Additional file 1: Figure S1) for all four phospholinked nucleotides used in SMRT reverse transcription. **Table S4.** Collapsed sequences of 16S rRNA and mRNA. **Table S5.** A list links to the raw data. Click here for file
