# Supplementary material for: Analysis of RNA base modification and structural rearrangement by single-molecule real-time detection of reverse transcription
Source: J Nanobiotechnology. 2013 Apr 3;11:8. doi: 10.1186/1477-3155-11-8 (PMC3623877; doi:10.1186/1477-3155-11-8)
Supplement: Additional file 1: Scheme S1 — Reaction mechanism of reverse transcriptase. Figure S1. Activity of HIV, AMV and MMLV reverse transcriptases (RTs) in the presence of phospholinked nucleotides (PL-Ns). Figure S2 (a)-(j). Examples of SMRT reverse transcription traces obtained with synthetic RNA template. Figure S3. Determination of binding, dissociation and incorporation rates for HIV RT using phospholinked nucleotides. Figure S4. Cumulative distributions of block widths during SMRT reverse transcription for incorporations of A (yellow), C (red), G (green), and T (blue) phospholinked nucleotides by HIV RT in ZMWs. Figure S5. Basepairing probabilities on mRNA template calculated using the Kinetic Trap Model (KTM). Figure S6. Basepairing probabilities on mRNA calculated using the Equilibrium Model (EM). Figure S7. Activity of RNA-dependent RNA polymerase from bacteriophage ϕ6 (ϕ6 RdRP) in the presence of a phospholinked rCTP. Table S1. RNA templates and DNA primers used in SMRT reverse transcription. Table S2. Sequences of DNA templates and corresponding DNA primers used in the bulk measurements of HIV RT transcription kinetics (Additional file 1: Figure S1). Table S3. Bulk kinetics data obtained with stopped-flow experiments (Additional file 1: Figure S1) for all four phospholinked nucleotides used in SMRT reverse transcription. Table S4. Collapsed sequences of 16S rRNA and mRNA. Table S5. A list links to the raw data. [file 1477-3155-11-8-S1.docx]

Supplementary Material for:

**Analysis of RNA base modification and structural rearrangement by single-molecule real-time detection of reverse transcription**

Igor D. Vilfan, Yu-Chih Tsai, Tyson A. Clark, Jeffrey Wegener, Qing Dai, Chengqi Yi, Tao Pan, Stephen W. Turner, Jonas Korlach


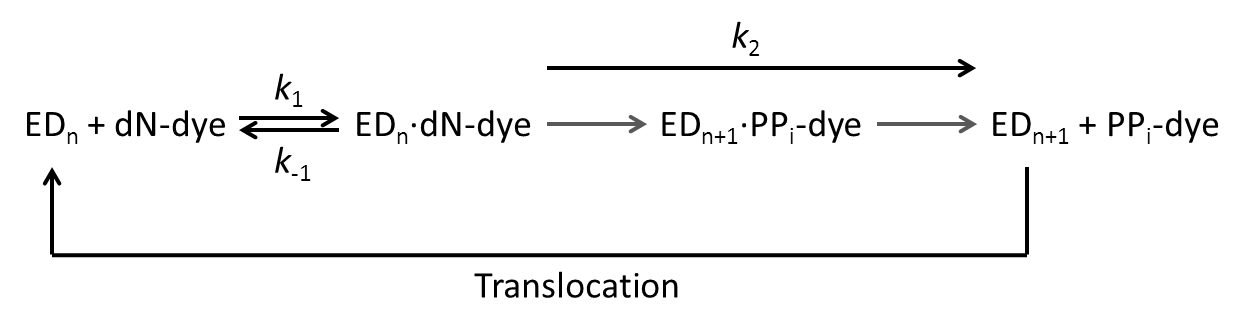


**Supplementary Scheme S1** Reaction mechanism of reverse transcriptase. The RT–RNA-cDNA complex (ED_n_) binds a complementary fluorescently labeled nucleotide (dN-dye) during the first step characterized by binding (*k*_1_) and dissociation (*k*_-1_) rates. The nucleotide binding event is followed by conformational changes and incorporation of the nucleotide in the growing cDNA strand (ED_n+1_), followed by fluorescently labeled pyrophosphate (PP_i_-dye) release. The latter two processes are described by an overall incorporation rate *k*_2_. After pyrophosphate release, the enzyme translocates to the next template position and repeats the reaction cycle.


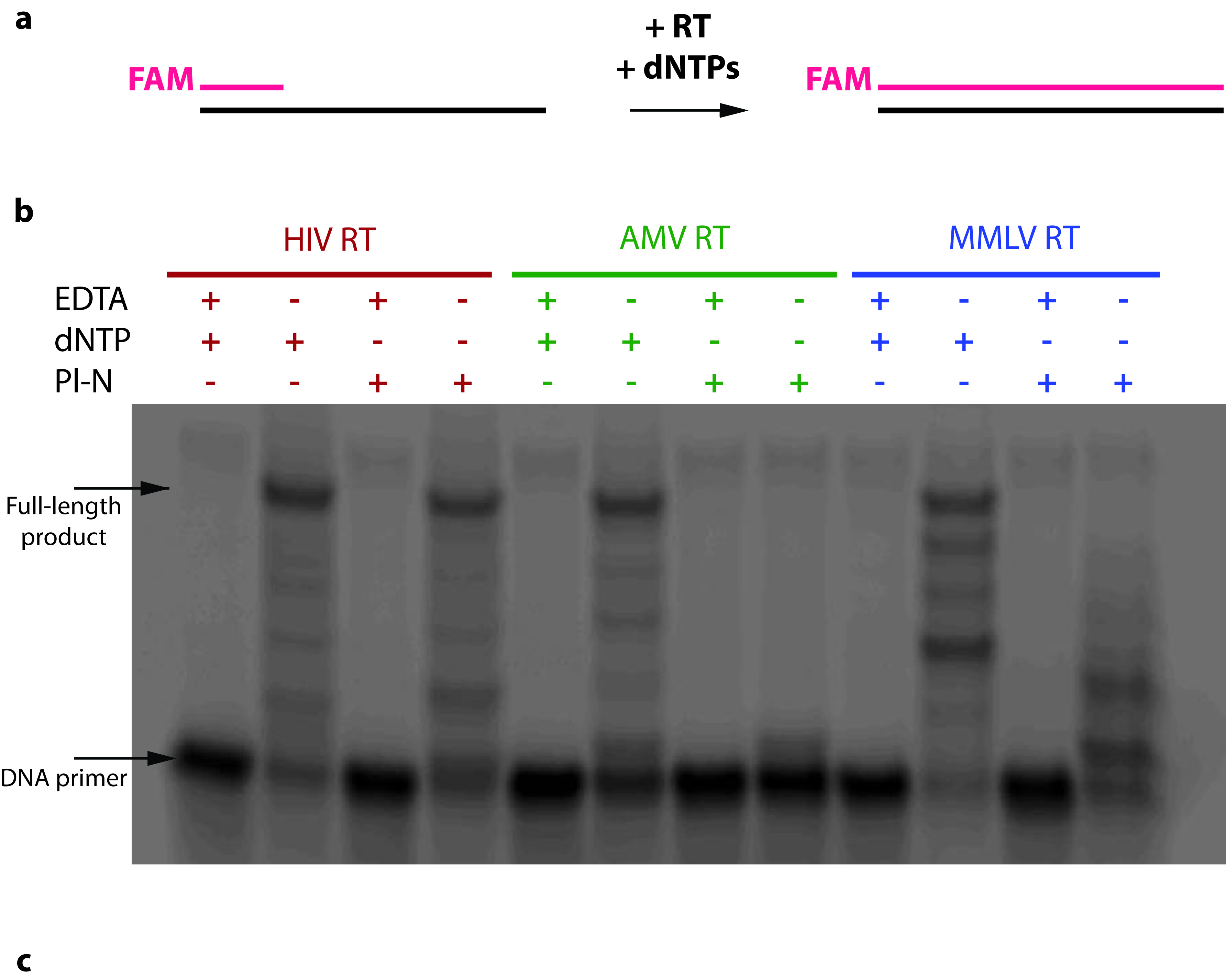


| **Reverse Transcriptase** | **HIV RT** | | **AMV RT** | | **MMLV RT** | |
| --- | --- | --- | --- | --- | --- | --- |
|  | dNTP | PL-N | dNTP | PL-N | dNTP | PL-N |
| ***% Template Utilized*** | 71.4 | 59.5 | 43.6 | 2.7 | 78.8 | 65.8 |
| ***% Full-length Product*** | 28.0 | 22.8 | 17.8 | ≈0 | 21.8 | ≈0 |

**Supplementary Figure S1** Activity of HIV, AMV and MMLV reverse transcriptases (RTs) in the presence of phospholinked nucleotides (PL-Ns). (**a**) FAM-labeled DNA primer (purple) was hybridized to synthetic RNA template (black) and the first-strand DNA synthesis was initiated by the addition of RT with native nucleotides (dNTPs) or PL-Ns (Supplementary Note S1, Online Methods). (**b**) Denaturing PAGE of the reverse transcriptions using HIV, AMV, and MMLV RTs in the presence of dNTPs and PL-Ns. (**c**) Activity of RTs in the presence of dNTPs and PL-Ns as determined from the denaturing PAGE in (b) and described in Supplementary Note S1.


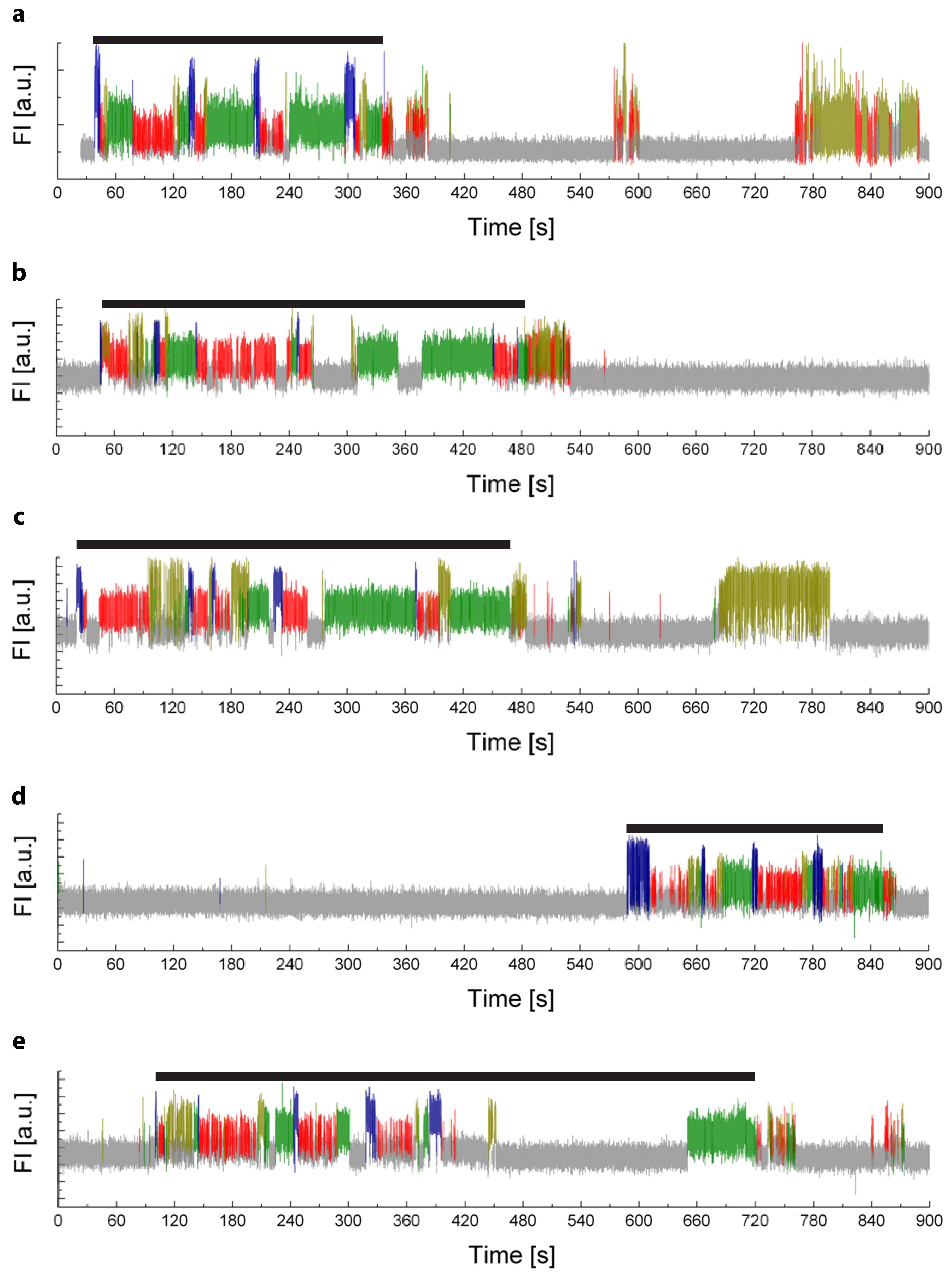


**
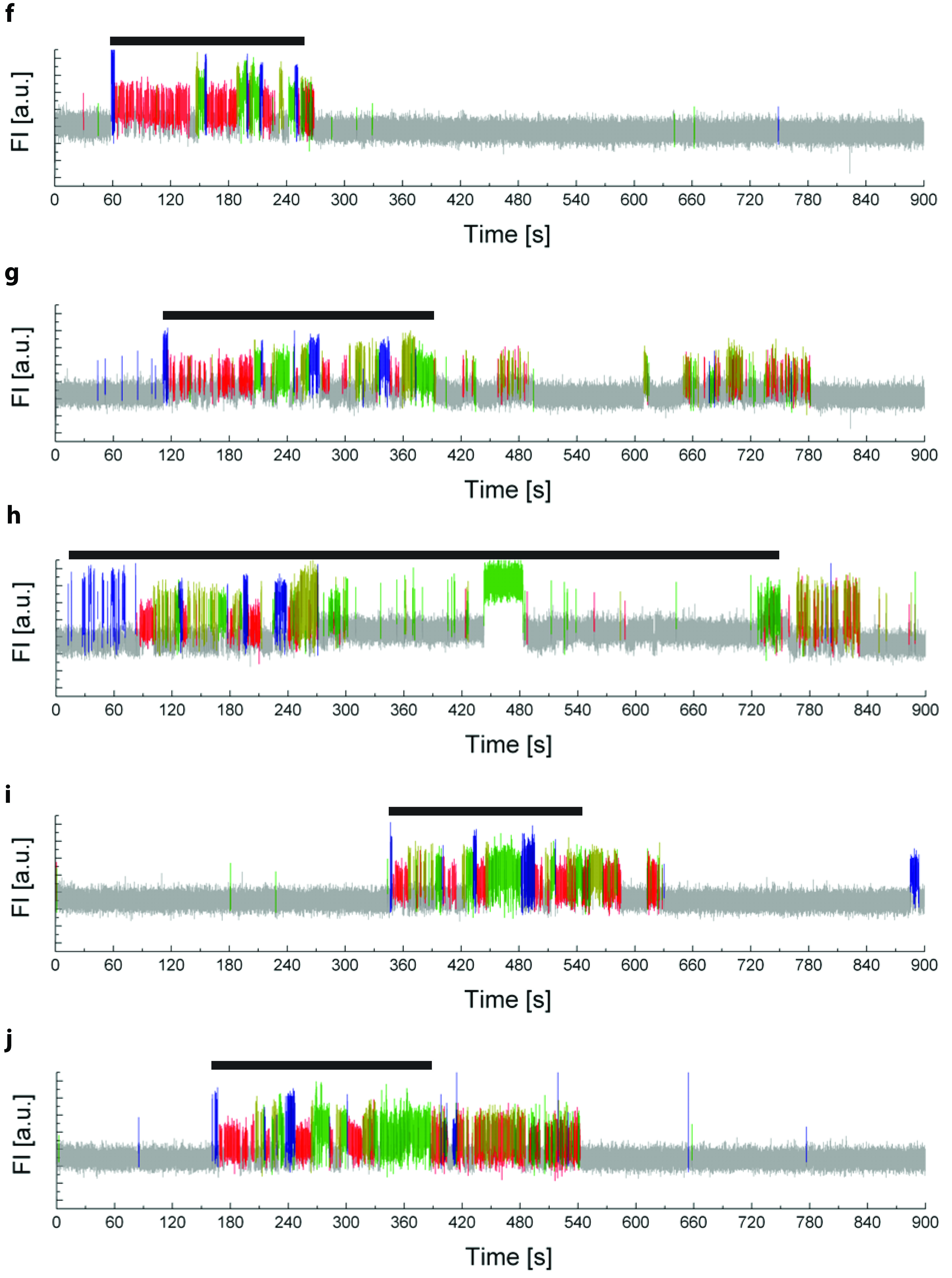
**

**Supplementary Figure S2** (**a**)-(**j**) Examples of SMRT reverse transcription traces obtained with synthetic RNA template. A section of each trace where reverse transcription of synthetic RNA template was detected is indicated with a solid black line. T, C, A, and G pulses are colored blue, red, dark yellow, and green, respectively. Example trace in (a) represents a full trace from Fig. 1c in the main text.


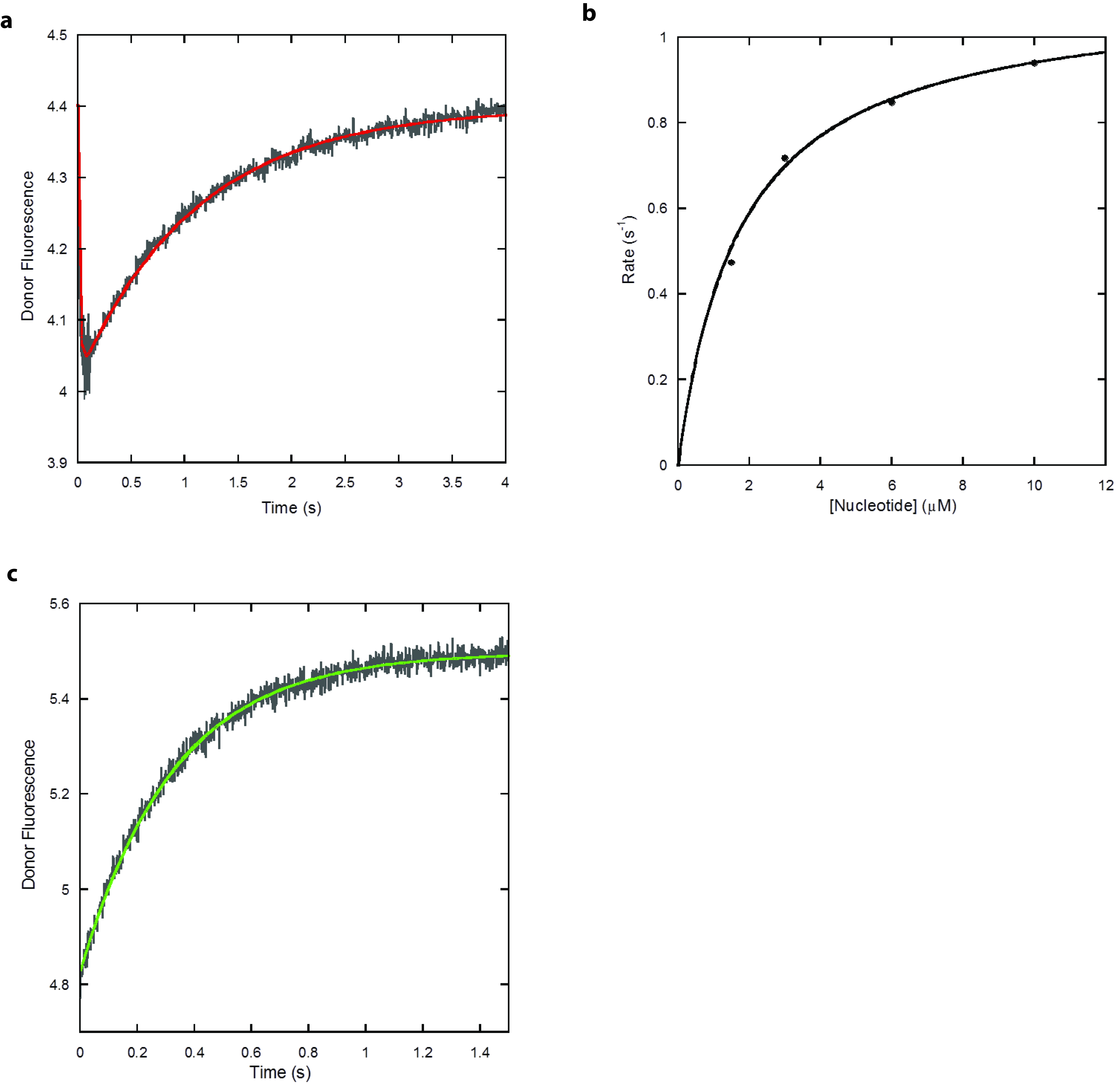


**Supplementary Figure S3** Determination of binding, dissociation and incorporation rates for HIV RT using phospholinked nucleotides. All the examples shown here are from the experiments with phospholinked deoxythymidine. (**a**) Stopped-flow experiment to determine nucleotide binding and incorporation rates (Material and Methods, Supplemental Scheme S1). Two transitions in FAM fluorescence can be identified. The initial decrease in FAM fluorescence was due to phospholinked nucleotide binding to the RT-DNA template complex which resulted in quenching of FAM fluorescence by the fluorophore on the nucleotide via fluorescence resonance energy transfer (FRET). Subsequently, the initial FAM fluorescence was recovered due to the release of the phospholinked fluorophore after nucleotide incorporation. The observed two-phase transitions can be related to the rates of nucleotide binding and pyrophosphate release following nucleotide incorporation. (**b**) Concentration dependence of the incorporation rates was used to determine *k*_2_. Similar concentration analysis was performed to obtain *k*_1_. (**c**) Increase in FAM fluorescence after dissociation of the phospholinked fluorescent nucleotide was used to determine nucleotide dissociation rate, *k_-1_* (Materials and Methods, Supplemental Scheme S1). Initially, FAM fluorescence was low due to quenching of FAM fluorescence by the phospholinked fluorophore on the nucleotide. The subsequent increase in FAM fluorescence upon addition of a large excess of unlabeled nucleotide resulted from the dissociation of the phospholinked fluorescent nucleotide from the ternary complex in the presence of excess unlabeled nucleotide and was used to determine *k*_-1_.


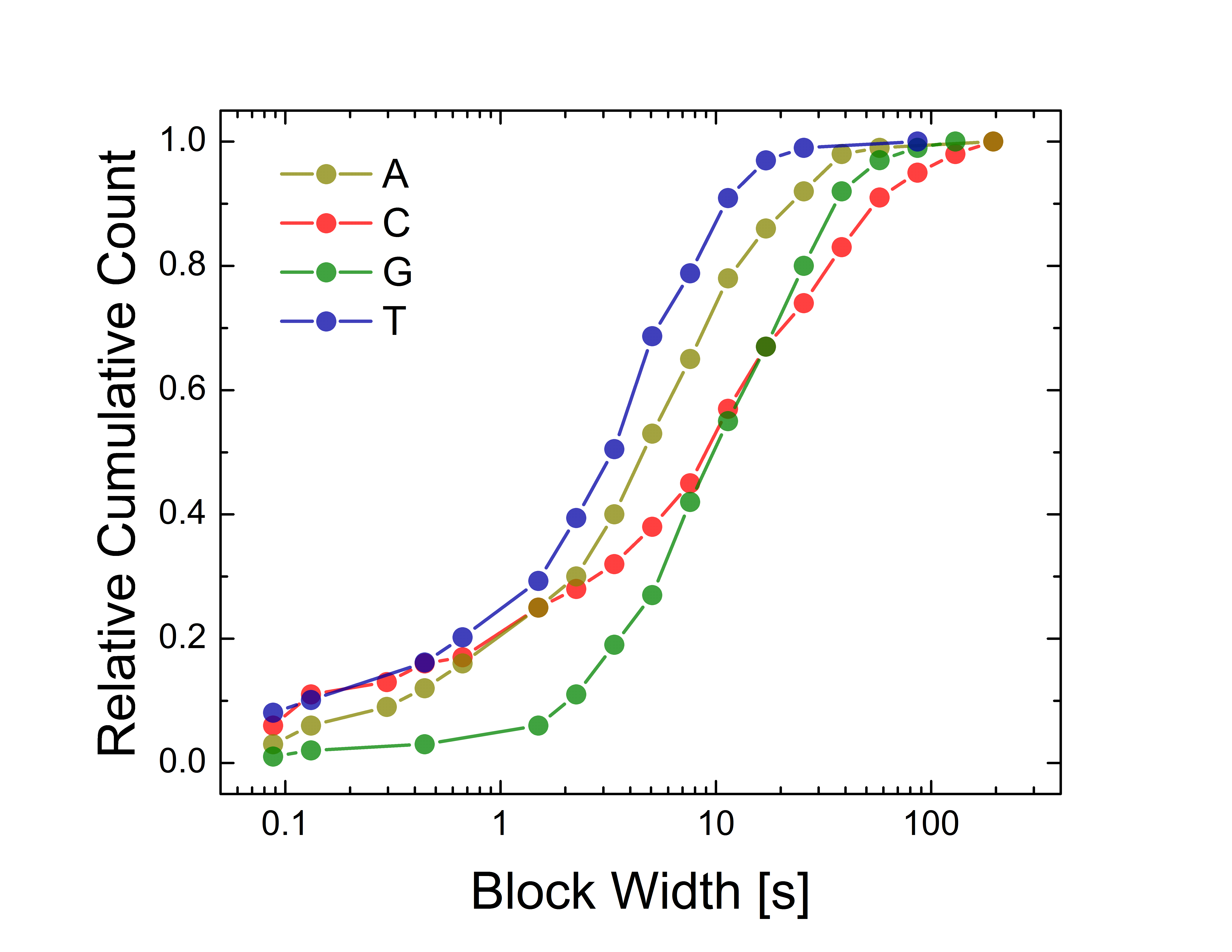


**Supplementary Figure S4** Cumulative distributions of block widths during SMRT reverse transcription for incorporations of A (yellow), C (red), G (green), and T (blue) phospholinked nucleotides by HIV RT in ZMWs.


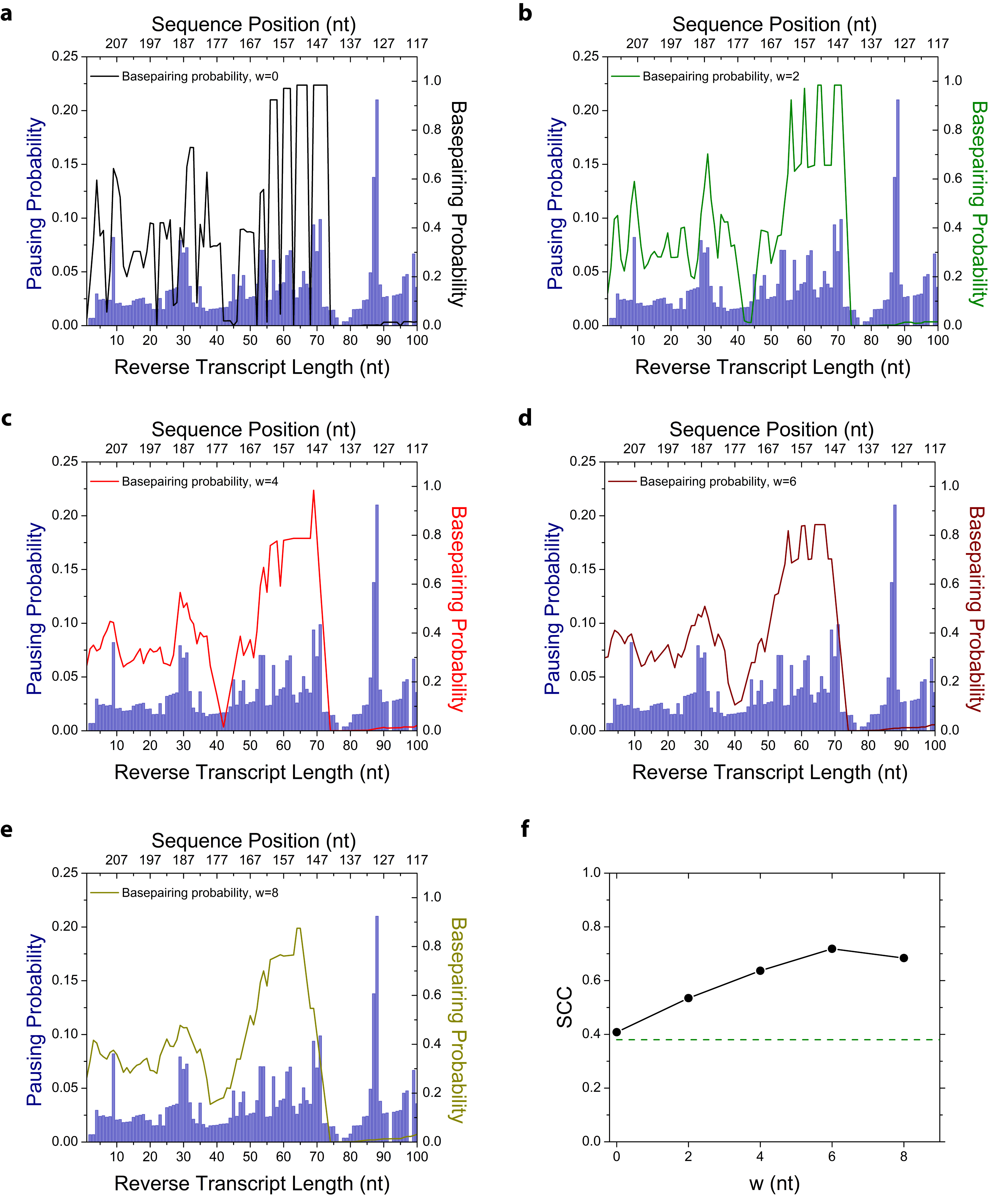


**Supplementary Figure S5** Basepairing probabilities on *mRNA* template calculated using the Kinetic Trap Model (KTM). Each graph contains two plots: (1) Pausing probability shown in blue bars, and (2) basepairing probabilities along the *mRNA* template as a colored line. The value of averaging parameter *w* was equal to: (**a**) *w* = 0, (**b**) *w* = 2, (**c**) *w* = 4, (**d**) *w* = 6, and (**e**) *w* = 8. (**f**) Spearman correlation coefficient (SCC) between pausing and basepairing probabilities for plots in (a) – (e). The critical value of SSC for the data set including the first 75 nucleotides of *mRNA* (N=75 at 99.9% reliability) is shown as a dashed green line.


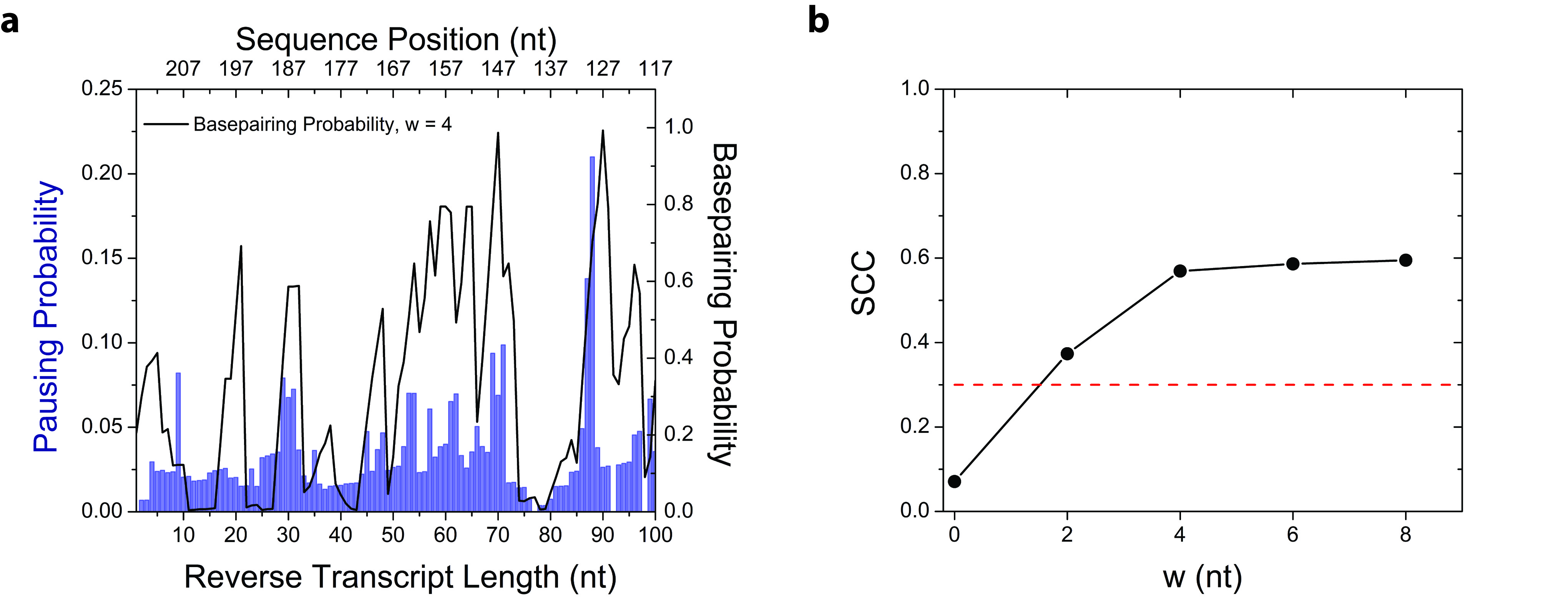


**Supplementary Figure S6** Basepairing probabilities on *mRNA* calculated using the Equilibrium Model (*EM*). (**a**) Comparison of pausing probability (blue bars) and basepairing probabilities (black line). Base pairing probabilities were calculated using *EM* and *w* equal to 0, 2, 4, 6, and 8. The plot contains only base pairing probabilities for *w* = 4. (**b**) Spearman correlation coefficient (SCC) for comparison of pausing probability and equilibrium basepairing probability as a function of parameter *w*. The critical value of SSC for the data set including the first 100 nucleotides of *mRNA* (N=100 at 99.9 % reliability) is shown as a dashed red line.


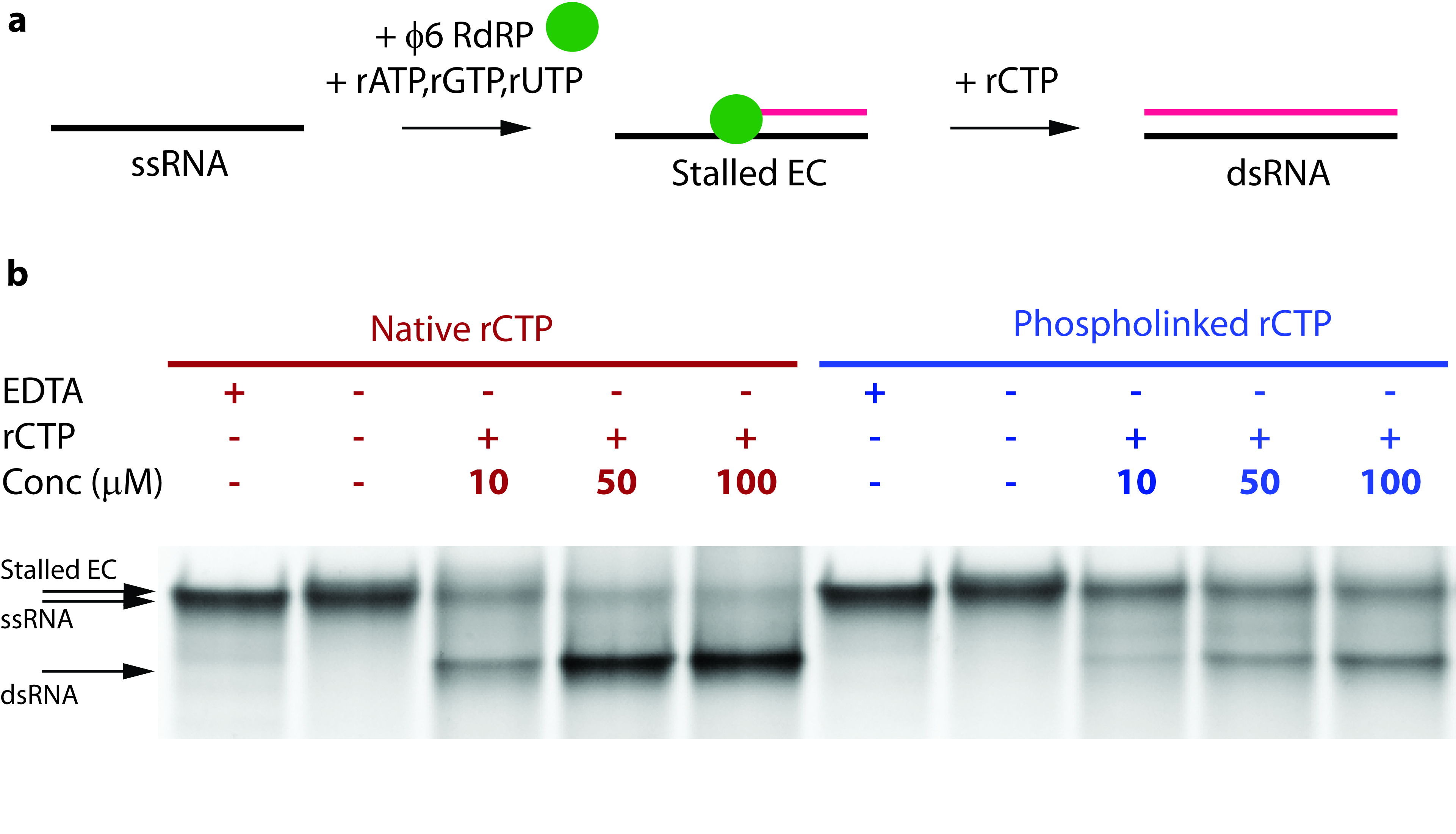


**Supplementary Figure S7** Activity of RNA-dependent RNA polymerase from bacteriophage φ6 (φ6 RdRP) in the presence of a phospholinked rCTP. (**a**) φ6 RdRP replication was first initiated in the presence of three ribonucleotides (rATP, rGTP, and rUTP) at a 3’-end of ssRNA template (Supplementary Table S1) resulting in the formation of an elongation complex (EC). EC stalled at the first G in the template due to the absence of rCTP. Upon the addition of the missing rCTP, the stalled EC resumed polymerization of the complementary strand to yield a double-stranded product (dsRNA). φ6 RdRP replication was carried out in the presence of native rCTP or phospholinked rCTP. (**b**) Native PAGE of φ6 RdRP replication reactions with native rCTP and phospholinked rCTP.

| **RNA Template Name** | **RNA Template Sequence**  **(5’-to’3’ direction)** | **DNA primer** |
| --- | --- | --- |
| *Synthetic RNA* | CUGACUGACUGACUGACUGA**GCGCGCUAUAGAUAUAAAAAGUGG** | *P5 or FAM-P5* |
| *m^6^A* | UUAG(m6A)CUCUGAACAAC**GCGCGCUAUAGAUAUAAAAAGUGG** | *P5* |
| *m^6^A Control* | UUAGACUCUGAACAAC**GCGCGCUAUAGAUAUAAAAAGUGG** | *P5* |
| *Native mRNA* | GGGUCAGAUCCGCUAGCGCUACCGGUCGCCACCAUGGUGAGCAAGGGCGCCGAGCUGUUCACCGGCAUCGUGCCCAUCCUGAUCGAGCUGAAUGGCGAUGUGAAUGGCCACAAGUUCAGCGUGAGCGGCGAGGGCGAGGGCGAUGCCACCUACGGCAAGCUGACCCUGAAGUUCAUCUGCACCACCGGCAAGCUGCCUGUGCCCUGGCCCACCCUGGUGACCACCCUGAGCUACGGCGUGCAGUGCUUCUCACGCUACCCCGAUCACAUGAAGCAGCACGACUUCUUCAAGAGCGCCAUGCCUGAGGGCUACAUCCAGGAGCGCACCAUCUUCUUCGAGGAUGACGGCAACUACAAGUCGCGCGCCGAGGUGAAGUUCGAGGGCGAUACCCUGGUGAAUCGCAUCGAGCUGACCGGCACCGAUUUCAAGGAGGAUGGCAACAUCCUGGGCAAUAAGAUGGAGUACAACUACAACGCCCACAAUGUGUACAUCAUGACCGACAAGGCCAAGAAUGGCAUCAAGGUGAACUUCAAGAUCCGCCACAACAUCGAGGAUGGCAGCGUGCAGCUGGCCGACCACUACCAGCAGAAUACCCCCAUCGGCGAUGGCCCUGUGCUGCUGCCCGAUAACCACUACCUGUCCACCCAGAGCGCCCUGUCCAAGGACCCCAACGAGAAGCGCGAUCACAUGAUCUACUUCGGCUUCGUGACCGCCGCCGCCAUCACCCACGGCAUGGAUGAGCUGUACAAGUCCGGACUCAGAUCUCGAGaCUGCUAAGCCCACAUUCCAUCCUAUCCAUUUCUGAGAUGGUUCUUAAUGAUCCAUUCCCUGGCAAACUUCUCUGAGCUUUAUAGCUUUGUAAUGCAUGCUUGGCUCUAAUGGGUUUCAUCUUAAAUAAAAACAG**A**CUCU**GUAGCGAUGUCAAAAUCUGCGCGCUA**UAGAUAUAAAAAGUUGGAUCCACCGGAUCUAGAUAACUGAUCAUAAUCAGCCAUACCACAUUUGUAGAGGUUUUACUUGCUUUAAAAAACCUCCCACACCUCCCCCUGAACCUGAAACAUAAAAUGAAUGCAAUUGUUGUUGUUAACUUGUUUAUUGCAGCUUAUAAUGGUUACAAAUAAAGCAAUAGCAUCACAAAUUUCACAAAUAAAGCAUUUUUUUCACUGCAAAAAAAAAA | *Pm6A* |
| *16S rRNA*  *(Only the first 200 nt at the 5’-end shown)* | AAAUUGAAGAGUUUGAUCAUGGCUCAGAUUGAACGCUGGCGGCAGGCCUAACACAUGCAAGUCGAACGGUAACAGGAAGCAGCUUGCUGCUUCGCUGACGAGUGGCGGACGGGUGAGUAA**UGUCUGGGAAACUGCCUGAUGGAGGG**GGAUAACUACUGGAAACGGUAGCUAAUACCGCAUAAUGUCGCAAGACCAAAGAG … | *P-rRNA* |
| *mRNA* | AGCGGCUUUGAUUCAGCAAGCCACAACAGUUAAAAACAAGGAUAUCAGGAAAUUUUUGGAUGGUAUCUAUGUCUCUGAAAGAGGAACUGUUCAGCAGGCUGAUGAAUAAGAUCUAAGAGUUACCUGGCUACAGAAAGAAGAUGCCAGAUGACACUUAAGACCUACUUGUGAUAUUUAAAUGAUGCAAUAAAAGACCUAUUGAUUUGGACCUUCUUC**UUAAAAAAAAAAAAAAAAAAAAGGGCGAAUUCG**UUUAAAC | *P-mRNA* |
| *ssRNA* | CAACCUAAAACUUACACACCCGGUAAGGAAAUAAAAAUGAAAUUUAAAUUCAAAUUCAAAUUC | *N/A* |
|  |  |  |
| **DNA Primer Name** | **DNA Primer Sequence**  **(5’-to-3’ direction)** |  |
| *P5* | Biotin-CCACTTTTTATATCTATAGCGCGC |  |
| *FAM-P5* | FAM-CCACTTTTTATATCTATAGCGCGC |  |
| *P-rRNA* | BiotinTEG-CCCTCCATCAGGCAGTTTCCCAGAC |  |
| *P-mRNA* | BiotinTEG-CGAATTCGCCCTTTTTTTTTTTTTTTTTTTTAA |  |
| *Pm6A* | BiotinTEG-TAGCGCGCAGATTTTGACATCGCTAC |  |

**Supplementary Table S1** RNA templates and DNA primers used in SMRT reverse transcription. Bolded parts of RNA sequences indicate DNA primer hybridization sites. (m6A) indicates N6-methyladenine. **A** indicates the predicted m6A position in the native mRNA. (N/A stands for ‘not applicable’.) (N/A = not applicable)

| **DNA Template Name** | **DNA Template Sequence** | **DNA Primer** |
| --- | --- | --- |
| Template A | FAM-AATACAGATCT**GACACCACCTACCACCTATC** | P-A or P-ddA |
| Template C | FAM-AATACG**GATCTGACACCACCTACCACCTATC** | P-C or P-ddC |
| Template G | FAM-AATATC**GATCTGACACCACCTACCACCTATC** | P-G or P-ddG |
| Template T | FAM-AATACA**GATCTGACACCACCTACCACCTATC** | P-T or P-ddT |
|  |  |  |
| **DNA Primer Name** | **DNA Primer Sequence** | **DNA Primer Group** |
| P-A | GATAGGTGGTAGGTGGTGTC | P |
| P-C | GATAGGTGGTAGGTGGTGTCAGATC |  |
| P-G | GATAGGTGGTAGGTGGTGTCAGATC |  |
| P-T | GATAGGTGGTAGGTGGTGTCAGATC |  |
| P-ddA | GATAGGTGGTAGGTGGTGTCdd | P-dd |
| P-ddC | GATAGGTGGTAGGTGGTGTCAGATCdd |  |
| P-ddG | GATAGGTGGTAGGTGGTGTCAGATCdd |  |
| P-ddT | GATAGGTGGTAGGTGGTGTCAGATCdd |  |

**Supplementary Table S2** Sequences of DNA templates and corresponding DNA primers used in the bulk measurements of HIV RT transcription kinetics (Supplemental Fig. S1). All DNA templates were labeled with 6-carboxyfluorescein (FAM) at their 5’-ends. The parts of DNA template sequences hybridizing to the corresponding primers have been bolded. DNA primers P-ddA, P-ddC, P-ddG, and P-ddT were dideoxy-terminated at their 3’-ends.


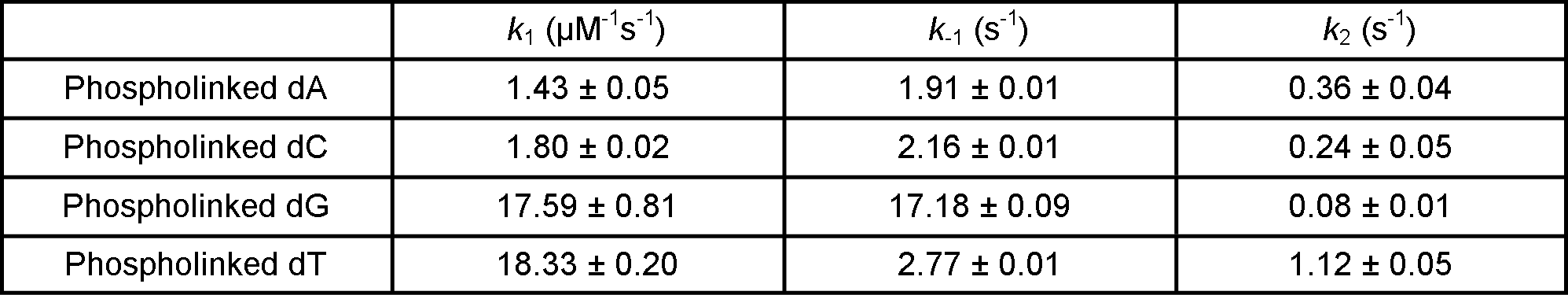


**Supplementary Table S3** Bulk kinetics data obtained with stopped-flow experiments (Supplementary Fig. S1) for all four phospholinked nucleotides used in SMRT reverse transcription. A comparison of rates *k*_1_, *k*_-1_, and *k*_2_ shows that the incorporation rates are slower than the nucleotide dissociation rates for all phospholinked nucleotides used in this study. This explains the multiple binding events (fluorescence pulses) observed in SMRT reverse transcription prior to nucleotide incorporation (Fig. 1d).

| **RNA Template Name** | **Collapsed Sequence**  **(NOTE: Only the sections that were SMRT reverse transcribed are shown.)** |
| --- | --- |
| *16S rRNA* | AUGAGAGUGAUCAUGCUCAGAUGACGCUGCGCAGCUACACAUGCAGUCGACGUACAGAGCAGCUGCUGCUCGCUGACGAGUGCGACGUGAGUA |
| *mRNA* | AGCGCUGAUCAGCAGCACACAGUACAGAUAUCAGAUGAUGUAUCUAUGUCUCUGAGAGACUGUCAGCAGCUGAUGAUAGAUCUAGAGUACUGCUACAGAGAGAUGCAGAUGACACUAGACUACUGUGAUAUAUGAUGCAUAGACUAUGAUGACUCUC |

**Supplementary Table S4** Collapsed sequences of 16S rRNA and mRNA. Only sections of the templates that were SMRT reverse transcribed were collapsed for the purpose of reverse transcript length determination (i.e., only the regions upstream of the DNA primer binding sequence in Supplementary Table S1).

| **RNA Template** | **Data Presented in …** | **Sequencing Run No.** | **Movie No.** | **Data Link** |
| --- | --- | --- | --- | --- |
| *Synthetic RNA* | **Figures 1c,d,e**  **Supplementary Figure S2** | 1 | **1** | [Pulse calling file](http://www.pacificbiosciences.com/devnet/files/datasets/publications/RNA_SmrtSeq/Synthetic_RNA_data/0885/ivilfan_SSB51829_new/m100721_151417_Geo_p1_b5.pls.h5) ; [Base calling file](http://www.pacificbiosciences.com/devnet/files/datasets/publications/RNA_SmrtSeq/Synthetic_RNA_data/0885/ivilfan_SSB51829_new/m100721_151417_Geo_p1_b5.bas.h5) |
|  |  | **2** | **1** | [Pulse calling file](http://www.pacificbiosciences.com/devnet/files/datasets/publications/RNA_SmrtSeq/Synthetic_RNA_data/0919/ivilfan_SSB51829/m100804_200759_Spa_p1_b5.pls.h5) ; [Base calling file](http://www.pacificbiosciences.com/devnet/files/datasets/publications/RNA_SmrtSeq/Synthetic_RNA_data/0919/ivilfan_SSB51829/m100804_200759_Spa_p1_b5.bas.h5) |
|  |  | **3** | **1** | [Pulse calling file](http://www.pacificbiosciences.com/devnet/files/datasets/publications/RNA_SmrtSeq/Synthetic_RNA_data/1369/ivilfan_SSB51829/m110309_182031_Twe_p1_b10.pls.h5) ; [Base calling file](http://www.pacificbiosciences.com/devnet/files/datasets/publications/RNA_SmrtSeq/Synthetic_RNA_data/1369/ivilfan_SSB51829/m110309_182031_Twe_p1_b10.bas.h5) |
| *m^6^A* | **Figures 2a,c** | **1** | **1** | [Pulse calling file](http://www.pacificbiosciences.com/devnet/files/datasets/publications/RNA_SmrtSeq/m6A_RNA_data/Synthetic_m6A_oligos/m6A_oligo/ivilfan_SSB51829/m100519_202104_Geo_p1_b5.pls.h5) ; [Base calling file](http://www.pacificbiosciences.com/devnet/files/datasets/publications/RNA_SmrtSeq/m6A_RNA_data/Synthetic_m6A_oligos/m6A_oligo/ivilfan_SSB51829/m100519_202104_Geo_p1_b5.pls.h5) |
|  |  | **2** | **1** | [Pulse calling file](http://www.pacificbiosciences.com/devnet/files/datasets/publications/RNA_SmrtSeq/m6A_RNA_data/Synthetic_m6A_oligos/m6A_oligo/ivilfan_SSB51829/m100519_204713_Geo_p1_b5.pls.h5) ; [Base calling file](http://www.pacificbiosciences.com/devnet/files/datasets/publications/RNA_SmrtSeq/m6A_RNA_data/Synthetic_m6A_oligos/m6A_oligo/ivilfan_SSB51829/m100519_204713_Geo_p1_b5.pls.h5) |
| *m^6^A Control* | **Figures 2b,d** | **1** | **1** | [Pulse calling file](http://www.pacificbiosciences.com/devnet/files/datasets/publications/RNA_SmrtSeq/m6A_RNA_data/Synthetic_m6A_oligos/Control_oligo/ivilfan_SSB51829/m100519_004523_Twe_p1_b5.pls.h5) ; [Base calling file](http://www.pacificbiosciences.com/devnet/files/datasets/publications/RNA_SmrtSeq/m6A_RNA_data/Synthetic_m6A_oligos/Control_oligo/ivilfan_SSB51829/m100519_004523_Twe_p1_b5.pls.h5) |
|  |  | **2** | **1** | [Pulse calling file](http://www.pacificbiosciences.com/devnet/files/datasets/publications/RNA_SmrtSeq/m6A_RNA_data/Synthetic_m6A_oligos/Control_oligo/0789/Reports_for_SeqPipeline/m100519_195426_Geo_p1_b5.pls.h5) ; [Base calling file](http://www.pacificbiosciences.com/devnet/files/datasets/publications/RNA_SmrtSeq/m6A_RNA_data/Synthetic_m6A_oligos/Control_oligo/919/Reports_for_SeqPipeline/m100519_195426_Geo_p1_b5.pls.h5) |
| *Native mRNA* | **Figures 3b,c** | **1** | **1** | [Pulse calling file](http://www.pacificbiosciences.com/devnet/files/datasets/publications/RNA_SmrtSeq/m6A_RNA_data/m6A_mRNA/Cellular_RNA/1547/ivilfan_SSB51829/m111108_155002_Geo_p1_b20.pls.h5) ; [Base calling file](http://www.pacificbiosciences.com/devnet/files/datasets/publications/RNA_SmrtSeq/m6A_RNA_data/m6A_mRNA/Cellular_RNA/1547/ivilfan_SSB51829/m111108_155002_Geo_p1_b20.bas.h5) |
|  |  | **2** | **1** | [Pulse calling file](http://www.pacificbiosciences.com/devnet/files/datasets/publications/RNA_SmrtSeq/m6A_RNA_data/m6A_mRNA/Cellular_RNA/1548/ivilfan_SSB51829/m111108_163150_Geo_p1_b25.pls.h5) ; [Base calling file](http://www.pacificbiosciences.com/devnet/files/datasets/publications/RNA_SmrtSeq/m6A_RNA_data/m6A_mRNA/Cellular_RNA/1548/ivilfan_SSB51829/m111108_163150_Geo_p1_b25.bas.h5) |
|  |  | **3** | **1** | [Pulse calling file](http://www.pacificbiosciences.com/devnet/files/datasets/publications/RNA_SmrtSeq/m6A_RNA_data/m6A_mRNA/Cellular_RNA/1549/ivilfan_SSB51829/m111108_171333_Geo_p1_b25.pls.h5) ; [Base calling file](http://www.pacificbiosciences.com/devnet/files/datasets/publications/RNA_SmrtSeq/m6A_RNA_data/m6A_mRNA/Cellular_RNA/1549/ivilfan_SSB51829/m111108_171333_Geo_p1_b25.bas.h5) |
|  |  | **4** | **1** | [Pulse calling file](http://www.pacificbiosciences.com/devnet/files/datasets/publications/RNA_SmrtSeq/m6A_RNA_data/m6A_mRNA/Cellular_RNA/1550/ivilfan_SSB51829/m111108_175542_Geo_p1_b25.pls.h5) ; [Base calling file](http://www.pacificbiosciences.com/devnet/files/datasets/publications/RNA_SmrtSeq/m6A_RNA_data/m6A_mRNA/Cellular_RNA/1550/ivilfan_SSB51829/m111108_175542_Geo_p1_b25.bas.h5) |
|  |  | **5** | **1** | [Pulse calling file](http://www.pacificbiosciences.com/devnet/files/datasets/publications/RNA_SmrtSeq/m6A_RNA_data/m6A_mRNA/Cellular_RNA/1551/ivilfan_SSB51829/m111108_183729_Geo_p1_b25.pls.h5) ; [Base calling file](http://www.pacificbiosciences.com/devnet/files/datasets/publications/RNA_SmrtSeq/m6A_RNA_data/m6A_mRNA/Cellular_RNA/1551/ivilfan_SSB51829/m111108_183729_Geo_p1_b25.bas.h5) |
|  |  | **6** | **1** | [Pulse calling file](http://www.pacificbiosciences.com/devnet/files/datasets/publications/RNA_SmrtSeq/m6A_RNA_data/m6A_mRNA/Cellular_RNA/1552/ivilfan_SSB51829/m111108_191936_Geo_p1_b25.pls.h5) ; [Base calling file](http://www.pacificbiosciences.com/devnet/files/datasets/publications/RNA_SmrtSeq/m6A_RNA_data/m6A_mRNA/Cellular_RNA/1552/ivilfan_SSB51829/m111108_191936_Geo_p1_b25.bas.h5) |
| *Native mRNA Control* | **Figures 3b,c** | **1** | **1** | [Pulse calling file](http://www.pacificbiosciences.com/devnet/files/datasets/publications/RNA_SmrtSeq/m6A_RNA_data/m6A_mRNA/Invitro_transcript/1553/ivilfan_SSB51829/m111108_200145_Geo_p1_b25.pls.h5) ; [Base calling file](http://www.pacificbiosciences.com/devnet/files/datasets/publications/RNA_SmrtSeq/m6A_RNA_data/m6A_mRNA/Invitro_transcript/1553/ivilfan_SSB51829/m111108_200145_Geo_p1_b25.bas.h5) |
|  |  | **2** | **1** | [Pulse calling file](http://www.pacificbiosciences.com/devnet/files/datasets/publications/RNA_SmrtSeq/m6A_RNA_data/m6A_mRNA/Invitro_transcript/1554/ivilfan_SSB51829/m111108_204250_Geo_p1_b25.pls.h5) ; [Base calling file](http://www.pacificbiosciences.com/devnet/files/datasets/publications/RNA_SmrtSeq/m6A_RNA_data/m6A_mRNA/Invitro_transcript/1554/ivilfan_SSB51829/m111108_204250_Geo_p1_b25.bas.h5) |
|  |  | **3** | **1** | [Pulse calling file](http://www.pacificbiosciences.com/devnet/files/datasets/publications/RNA_SmrtSeq/m6A_RNA_data/m6A_mRNA/Invitro_transcript/1555/ivilfan_SSB51829/m111108_212059_Geo_p1_b25.pls.h5) ; [Base calling file](http://www.pacificbiosciences.com/devnet/files/datasets/publications/RNA_SmrtSeq/m6A_RNA_data/m6A_mRNA/Invitro_transcript/1555/ivilfan_SSB51829/m111108_212059_Geo_p1_b25.bas.h5) |
|  |  | **4** | **1** | [Pulse calling file](http://www.pacificbiosciences.com/devnet/files/datasets/publications/RNA_SmrtSeq/m6A_RNA_data/m6A_mRNA/Invitro_transcript/1556/ivilfan_SSB51829/m111108_220024_Geo_p1_b25.pls.h5) ; [Base calling file](http://www.pacificbiosciences.com/devnet/files/datasets/publications/RNA_SmrtSeq/m6A_RNA_data/m6A_mRNA/Invitro_transcript/1556/ivilfan_SSB51829/m111108_220024_Geo_p1_b25.bas.h5) |
| *16S rRNA* | **Figure 4a** | **1** | **1** | [Pulse calling file](http://www.pacificbiosciences.com/devnet/files/datasets/publications/RNA_SmrtSeq/16S_rRNA_data/1350/ivilfan_SSB51829/m110211_174823_Spa_p1_b20.pls.h5) ; [Base calling file](http://www.pacificbiosciences.com/devnet/files/datasets/publications/RNA_SmrtSeq/16S_rRNA_data/1350/ivilfan_SSB51829/m110211_174823_Spa_p1_b20.bas.h5) |
|  |  |  | **2** | [Pulse calling file](http://www.pacificbiosciences.com/devnet/files/datasets/publications/RNA_SmrtSeq/16S_rRNA_data/1350/ivilfan_SSB51829/m110211_175522_Spa_p2_b20.pls.h5) ; [Base calling file](http://www.pacificbiosciences.com/devnet/files/datasets/publications/RNA_SmrtSeq/16S_rRNA_data/1350/ivilfan_SSB51829/m110211_175522_Spa_p2_b20.bas.h5) |
|  |  |  | **3** | [Pulse calling file](http://www.pacificbiosciences.com/devnet/files/datasets/publications/RNA_SmrtSeq/16S_rRNA_data/1350/ivilfan_SSB51829/m110211_180215_Spa_p3_b20.pls.h5) ; [Base calling file](http://www.pacificbiosciences.com/devnet/files/datasets/publications/RNA_SmrtSeq/16S_rRNA_data/1350/ivilfan_SSB51829/m110211_180215_Spa_p3_b20.bas.h5) |
|  |  |  | **4** | [Pulse calling file](http://www.pacificbiosciences.com/devnet/files/datasets/publications/RNA_SmrtSeq/16S_rRNA_data/1350/ivilfan_SSB51829/m110211_180912_Spa_p4_b20.pls.h5) ; [Base calling file](http://www.pacificbiosciences.com/devnet/files/datasets/publications/RNA_SmrtSeq/16S_rRNA_data/1350/ivilfan_SSB51829/m110211_180912_Spa_p4_b20.bas.h5) |
|  |  |  | **5** | [Pulse calling file](http://www.pacificbiosciences.com/devnet/files/datasets/publications/RNA_SmrtSeq/16S_rRNA_data/1350/ivilfan_SSB51829/m110211_181609_Spa_p5_b20.pls.h5) ; [Base calling file](http://www.pacificbiosciences.com/devnet/files/datasets/publications/RNA_SmrtSeq/16S_rRNA_data/1350/ivilfan_SSB51829/m110211_181609_Spa_p5_b20.bas.h5) |
|  |  |  | **6** | [Pulse calling file](http://www.pacificbiosciences.com/devnet/files/datasets/publications/RNA_SmrtSeq/16S_rRNA_data/1350/ivilfan_SSB51829/m110211_183326_Spa_p6_b20.pls.h5) ; [Base calling file](http://www.pacificbiosciences.com/devnet/files/datasets/publications/RNA_SmrtSeq/16S_rRNA_data/1350/ivilfan_SSB51829/m110211_183326_Spa_p6_b20.bas.h5) |
|  |  |  | **7** | [Pulse calling file](http://www.pacificbiosciences.com/devnet/files/datasets/publications/RNA_SmrtSeq/16S_rRNA_data/1350/ivilfan_SSB51829/m110211_184025_Spa_p7_b20.pls.h5) ; [Base calling file](http://www.pacificbiosciences.com/devnet/files/datasets/publications/RNA_SmrtSeq/16S_rRNA_data/1350/ivilfan_SSB51829/m110211_184025_Spa_p7_b20.bas.h5) |
|  |  |  | **8** | [Pulse calling file](http://www.pacificbiosciences.com/devnet/files/datasets/publications/RNA_SmrtSeq/16S_rRNA_data/1350/ivilfan_SSB51829/m110211_184711_Spa_p8_b20.pls.h5) ; [Base calling file](http://www.pacificbiosciences.com/devnet/files/datasets/publications/RNA_SmrtSeq/16S_rRNA_data/1350/ivilfan_SSB51829/m110211_184711_Spa_p8_b20.bas.h5) |
|  |  |  | **9** | [Pulse calling file](http://www.pacificbiosciences.com/devnet/files/datasets/publications/RNA_SmrtSeq/16S_rRNA_data/1350/ivilfan_SSB51829/m110211_185418_Spa_p9_b20.pls.h5) ; [Base calling file](http://www.pacificbiosciences.com/devnet/files/datasets/publications/RNA_SmrtSeq/16S_rRNA_data/1350/ivilfan_SSB51829/m110211_185418_Spa_p9_b20.bas.h5) |
|  |  |  | **10** | [Pulse calling file](http://www.pacificbiosciences.com/devnet/files/datasets/publications/RNA_SmrtSeq/16S_rRNA_data/1350/ivilfan_SSB51829/m110211_190107_Spa_p10_b20.pls.h5) ; [Base calling file](http://www.pacificbiosciences.com/devnet/files/datasets/publications/RNA_SmrtSeq/16S_rRNA_data/1350/ivilfan_SSB51829/m110211_190107_Spa_p10_b20.bas.h5) |
|  |  | **2** | **1** | [Pulse calling file](http://www.pacificbiosciences.com/devnet/files/datasets/publications/RNA_SmrtSeq/16S_rRNA_data/1351/ivilfan_SSB51829/m110211_192854_Spa_p1_b20.pls.h5) ; [Base calling file](http://www.pacificbiosciences.com/devnet/files/datasets/publications/RNA_SmrtSeq/16S_rRNA_data/1351/ivilfan_SSB51829/m110211_192854_Spa_p1_b20.bas.h5) |
|  |  |  | **2** | [Pulse calling file](http://www.pacificbiosciences.com/devnet/files/datasets/publications/RNA_SmrtSeq/16S_rRNA_data/1351/ivilfan_SSB51829/m110211_193603_Spa_p2_b20.pls.h5) ; [Base calling file](http://www.pacificbiosciences.com/devnet/files/datasets/publications/RNA_SmrtSeq/16S_rRNA_data/1351/ivilfan_SSB51829/m110211_193603_Spa_p2_b20.bas.h5) |
|  |  |  | **3** | [Pulse calling file](http://www.pacificbiosciences.com/devnet/files/datasets/publications/RNA_SmrtSeq/16S_rRNA_data/1351/ivilfan_SSB51829/m110211_194243_Spa_p3_b20.pls.h5) ; [Base calling file](http://www.pacificbiosciences.com/devnet/files/datasets/publications/RNA_SmrtSeq/16S_rRNA_data/1351/ivilfan_SSB51829/m110211_194243_Spa_p3_b20.bas.h5) |
|  |  |  | **4** | [Pulse calling file](http://www.pacificbiosciences.com/devnet/files/datasets/publications/RNA_SmrtSeq/16S_rRNA_data/1351/ivilfan_SSB51829/m110211_194936_Spa_p4_b20.pls.h5) ; [Base calling file](http://www.pacificbiosciences.com/devnet/files/datasets/publications/RNA_SmrtSeq/16S_rRNA_data/1351/ivilfan_SSB51829/m110211_194936_Spa_p4_b20.bas.h5) |
|  |  |  | **5** | [Pulse calling file](http://www.pacificbiosciences.com/devnet/files/datasets/publications/RNA_SmrtSeq/16S_rRNA_data/1351/ivilfan_SSB51829/m110211_195704_Spa_p5_b20.pls.h5) ; [Base calling file](http://www.pacificbiosciences.com/devnet/files/datasets/publications/RNA_SmrtSeq/16S_rRNA_data/1351/ivilfan_SSB51829/m110211_195704_Spa_p5_b20.bas.h5) |
|  |  |  | **6** | [Pulse calling file](http://www.pacificbiosciences.com/devnet/files/datasets/publications/RNA_SmrtSeq/16S_rRNA_data/1351/ivilfan_SSB51829/m110211_201447_Spa_p6_b20.pls.h5) ; [Base calling file](http://www.pacificbiosciences.com/devnet/files/datasets/publications/RNA_SmrtSeq/16S_rRNA_data/1351/ivilfan_SSB51829/m110211_201447_Spa_p6_b20.bas.h5) |
|  |  |  | **7** | [Pulse calling file](http://www.pacificbiosciences.com/devnet/files/datasets/publications/RNA_SmrtSeq/16S_rRNA_data/1351/ivilfan_SSB51829/m110211_202151_Spa_p7_b20.pls.h5) ; [Base calling file](http://www.pacificbiosciences.com/devnet/files/datasets/publications/RNA_SmrtSeq/16S_rRNA_data/1351/ivilfan_SSB51829/m110211_202151_Spa_p7_b20.bas.h5) |
|  |  |  | **8** | [Pulse calling file](http://www.pacificbiosciences.com/devnet/files/datasets/publications/RNA_SmrtSeq/16S_rRNA_data/1351/ivilfan_SSB51829/m110211_202924_Spa_p8_b20.pls.h5) ; [Base calling file](http://www.pacificbiosciences.com/devnet/files/datasets/publications/RNA_SmrtSeq/16S_rRNA_data/1351/ivilfan_SSB51829/m110211_202924_Spa_p8_b20.bas.h5) |
|  |  |  | **9** | [Pulse calling file](http://www.pacificbiosciences.com/devnet/files/datasets/publications/RNA_SmrtSeq/16S_rRNA_data/1351/ivilfan_SSB51829/m110211_203647_Spa_p9_b20.pls.h5) ; [Base calling file](http://www.pacificbiosciences.com/devnet/files/datasets/publications/RNA_SmrtSeq/16S_rRNA_data/1351/ivilfan_SSB51829/m110211_203647_Spa_p9_b20.bas.h5) |
|  |  |  | **10** | [Pulse calling file](http://www.pacificbiosciences.com/devnet/files/datasets/publications/RNA_SmrtSeq/16S_rRNA_data/1351/ivilfan_SSB51829/m110211_204411_Spa_p10_b20.pls.h5) ; [Base calling file](http://www.pacificbiosciences.com/devnet/files/datasets/publications/RNA_SmrtSeq/16S_rRNA_data/1351/ivilfan_SSB51829/m110211_204411_Spa_p10_b20.bas.h5) |
| *mRNA* | **Figure 4b** | **1** | **1** | [Pulse calling file](http://www.pacificbiosciences.com/devnet/files/datasets/publications/RNA_SmrtSeq/mRNA_data/1338/ivilfan_SSB51829/m110204_190308_Spa_p1_b20.pls.h5) ; [Base calling file](http://www.pacificbiosciences.com/devnet/files/datasets/publications/RNA_SmrtSeq/mRNA_data/1338/ivilfan_SSB51829/m110204_190308_Spa_p1_b20.bas.h5) |
|  |  |  | **2** | [Pulse calling file](http://www.pacificbiosciences.com/devnet/files/datasets/publications/RNA_SmrtSeq/mRNA_data/1338/ivilfan_SSB51829/m110204_191018_Spa_p2_b20.pls.h5) ; [Base calling file](http://www.pacificbiosciences.com/devnet/files/datasets/publications/RNA_SmrtSeq/mRNA_data/1338/ivilfan_SSB51829/m110204_191018_Spa_p2_b20.bas.h5) |
|  |  |  | **3** | [Pulse calling file](http://www.pacificbiosciences.com/devnet/files/datasets/publications/RNA_SmrtSeq/mRNA_data/1338/ivilfan_SSB51829/m110204_191645_Spa_p3_b20.pls.h5) ; [Base calling file](http://www.pacificbiosciences.com/devnet/files/datasets/publications/RNA_SmrtSeq/mRNA_data/1338/ivilfan_SSB51829/m110204_191645_Spa_p3_b20.bas.h5) |
|  |  |  | **4** | [Pulse calling file](http://www.pacificbiosciences.com/devnet/files/datasets/publications/RNA_SmrtSeq/mRNA_data/1338/ivilfan_SSB51829/m110204_192340_Spa_p4_b20.pls.h5) ; [Base calling file](http://www.pacificbiosciences.com/devnet/files/datasets/publications/RNA_SmrtSeq/mRNA_data/1338/ivilfan_SSB51829/m110204_192340_Spa_p4_b20.bas.h5) |
|  |  |  | **5** | [Pulse calling file](http://www.pacificbiosciences.com/devnet/files/datasets/publications/RNA_SmrtSeq/mRNA_data/1338/ivilfan_SSB51829/m110204_193031_Spa_p5_b20.pls.h5) ; [Base calling file](http://www.pacificbiosciences.com/devnet/files/datasets/publications/RNA_SmrtSeq/mRNA_data/1338/ivilfan_SSB51829/m110204_193031_Spa_p5_b20.bas.h5) |
|  |  |  | **6** | [Pulse calling file](http://www.pacificbiosciences.com/devnet/files/datasets/publications/RNA_SmrtSeq/mRNA_data/1338/ivilfan_SSB51829/m110204_194930_Spa_p6_b20.pls.h5) ; [Base calling file](http://www.pacificbiosciences.com/devnet/files/datasets/publications/RNA_SmrtSeq/mRNA_data/1338/ivilfan_SSB51829/m110204_194930_Spa_p6_b20.bas.h5) |
|  |  |  | **7** | [Pulse calling file](http://www.pacificbiosciences.com/devnet/files/datasets/publications/RNA_SmrtSeq/mRNA_data/1338/ivilfan_SSB51829/m110204_195654_Spa_p7_b20.pls.h5) ; [Base calling file](http://www.pacificbiosciences.com/devnet/files/datasets/publications/RNA_SmrtSeq/mRNA_data/1338/ivilfan_SSB51829/m110204_195654_Spa_p7_b20.bas.h5) |
|  |  |  | **8** | [Pulse calling file](http://www.pacificbiosciences.com/devnet/files/datasets/publications/RNA_SmrtSeq/mRNA_data/1338/ivilfan_SSB51829/m110204_200404_Spa_p8_b20.pls.h5) ; [Base calling file](http://www.pacificbiosciences.com/devnet/files/datasets/publications/RNA_SmrtSeq/mRNA_data/1338/ivilfan_SSB51829/m110204_200404_Spa_p8_b20.bas.h5) |
|  |  |  | **9** | [Pulse calling file](http://www.pacificbiosciences.com/devnet/files/datasets/publications/RNA_SmrtSeq/mRNA_data/1338/ivilfan_SSB51829/m110204_201102_Spa_p9_b20.pls.h5) ; [Base calling file](http://www.pacificbiosciences.com/devnet/files/datasets/publications/RNA_SmrtSeq/mRNA_data/1338/ivilfan_SSB51829/m110204_201102_Spa_p9_b20.bas.h5) |
|  |  |  | **10** | [Pulse calling file](http://www.pacificbiosciences.com/devnet/files/datasets/publications/RNA_SmrtSeq/mRNA_data/1338/ivilfan_SSB51829/m110204_201748_Spa_p10_b20.pls.h5) ; [Base calling file](http://www.pacificbiosciences.com/devnet/files/datasets/publications/RNA_SmrtSeq/mRNA_data/1338/ivilfan_SSB51829/m110204_201748_Spa_p10_b20.bas.h5) |
|  |  | **2** | **1** | [Pulse calling file](http://www.pacificbiosciences.com/devnet/files/datasets/publications/RNA_SmrtSeq/mRNA_data/1358/ivilfan_SSB51829/m110218_165719_Spa_p1_b15.pls.h5) ; [Base calling file](http://www.pacificbiosciences.com/devnet/files/datasets/publications/RNA_SmrtSeq/mRNA_data/1358/ivilfan_SSB51829/m110218_165719_Spa_p1_b15.bas.h5) |
|  |  |  | **2** | [Pulse calling file](http://www.pacificbiosciences.com/devnet/files/datasets/publications/RNA_SmrtSeq/mRNA_data/1358/ivilfan_SSB51829/m110218_170438_Spa_p2_b15.pls.h5) ; [Base calling file](http://www.pacificbiosciences.com/devnet/files/datasets/publications/RNA_SmrtSeq/mRNA_data/1358/ivilfan_SSB51829/m110218_170438_Spa_p2_b15.bas.h5) |
|  |  |  | **3** | [Pulse calling file](http://www.pacificbiosciences.com/devnet/files/datasets/publications/RNA_SmrtSeq/mRNA_data/1358/ivilfan_SSB51829/m110218_171224_Spa_p3_b15.pls.h5) ; [Base calling file](http://www.pacificbiosciences.com/devnet/files/datasets/publications/RNA_SmrtSeq/mRNA_data/1358/ivilfan_SSB51829/m110218_171224_Spa_p3_b15.bas.h5) |
|  |  |  | **4** | [Pulse calling file](http://www.pacificbiosciences.com/devnet/files/datasets/publications/RNA_SmrtSeq/mRNA_data/1358/ivilfan_SSB51829/m110218_171951_Spa_p4_b15.pls.h5) ; [Base calling file](http://www.pacificbiosciences.com/devnet/files/datasets/publications/RNA_SmrtSeq/mRNA_data/1358/ivilfan_SSB51829/m110218_171951_Spa_p4_b15.bas.h5) |
|  |  |  | **5** | [Pulse calling file](http://www.pacificbiosciences.com/devnet/files/datasets/publications/RNA_SmrtSeq/mRNA_data/1358/ivilfan_SSB51829/m110218_172629_Spa_p5_b15.pls.h5) ; [Base calling file](http://www.pacificbiosciences.com/devnet/files/datasets/publications/RNA_SmrtSeq/mRNA_data/1358/ivilfan_SSB51829/m110218_172629_Spa_p5_b15.bas.h5) |
|  |  |  | **6** | [Pulse calling file](http://www.pacificbiosciences.com/devnet/files/datasets/publications/RNA_SmrtSeq/mRNA_data/1358/ivilfan_SSB51829/m110218_174606_Spa_p6_b15.pls.h5) ; [Base calling file](http://www.pacificbiosciences.com/devnet/files/datasets/publications/RNA_SmrtSeq/mRNA_data/1358/ivilfan_SSB51829/m110218_174606_Spa_p6_b15.bas.h5) |
|  |  |  | **7** | [Pulse calling file](http://www.pacificbiosciences.com/devnet/files/datasets/publications/RNA_SmrtSeq/mRNA_data/1358/ivilfan_SSB51829/m110218_175354_Spa_p7_b15.pls.h5) ; [Base calling file](http://www.pacificbiosciences.com/devnet/files/datasets/publications/RNA_SmrtSeq/mRNA_data/1358/ivilfan_SSB51829/m110218_175354_Spa_p7_b15.bas.h5) |
|  |  |  | **8** | [Pulse calling file](http://www.pacificbiosciences.com/devnet/files/datasets/publications/RNA_SmrtSeq/mRNA_data/1358/ivilfan_SSB51829/m110218_180040_Spa_p8_b15.pls.h5) ; [Base calling file](http://www.pacificbiosciences.com/devnet/files/datasets/publications/RNA_SmrtSeq/mRNA_data/1358/ivilfan_SSB51829/m110218_180040_Spa_p8_b15.bas.h5) |
|  |  |  | **9** | [Pulse calling file](http://www.pacificbiosciences.com/devnet/files/datasets/publications/RNA_SmrtSeq/mRNA_data/1358/ivilfan_SSB51829/m110218_180759_Spa_p9_b15.pls.h5) ; [Base calling file](http://www.pacificbiosciences.com/devnet/files/datasets/publications/RNA_SmrtSeq/mRNA_data/1358/ivilfan_SSB51829/m110218_180759_Spa_p9_b15.bas.h5) |
|  |  |  | **10** | [Pulse calling file](http://www.pacificbiosciences.com/devnet/files/datasets/publications/RNA_SmrtSeq/mRNA_data/1358/ivilfan_SSB51829/m110218_181439_Spa_p10_b15.pls.h5) ; [Base calling file](http://www.pacificbiosciences.com/devnet/files/datasets/publications/RNA_SmrtSeq/mRNA_data/1358/ivilfan_SSB51829/m110218_181439_Spa_p10_b15.bas.h5) |
|  |  | **3** | **1** | [Pulse calling file](http://www.pacificbiosciences.com/devnet/files/datasets/publications/RNA_SmrtSeq/mRNA_data/1359/ivilfan_SSB51829/m110218_183738_Spa_p1_b15.pls.h5) ; [Base calling file](http://www.pacificbiosciences.com/devnet/files/datasets/publications/RNA_SmrtSeq/mRNA_data/1359/ivilfan_SSB51829/m110218_183738_Spa_p1_b15.bas.h5) |
|  |  |  | **2** | [Pulse calling file](http://www.pacificbiosciences.com/devnet/files/datasets/publications/RNA_SmrtSeq/mRNA_data/1359/ivilfan_SSB51829/m110218_184430_Spa_p2_b15.pls.h5) ; [Base calling file](http://www.pacificbiosciences.com/devnet/files/datasets/publications/RNA_SmrtSeq/mRNA_data/1359/ivilfan_SSB51829/m110218_184430_Spa_p2_b15.bas.h5) |
|  |  |  | **3** | [Pulse calling file](http://www.pacificbiosciences.com/devnet/files/datasets/publications/RNA_SmrtSeq/mRNA_data/1359/ivilfan_SSB51829/m110218_185117_Spa_p3_b15.pls.h5) ; [Base calling file](http://www.pacificbiosciences.com/devnet/files/datasets/publications/RNA_SmrtSeq/mRNA_data/1359/ivilfan_SSB51829/m110218_185117_Spa_p3_b15.bas.h5) |
|  |  |  | **4** | [Pulse calling file](http://www.pacificbiosciences.com/devnet/files/datasets/publications/RNA_SmrtSeq/mRNA_data/1359/ivilfan_SSB51829/m110218_185803_Spa_p4_b15.pls.h5) ; [Base calling file](http://www.pacificbiosciences.com/devnet/files/datasets/publications/RNA_SmrtSeq/mRNA_data/1359/ivilfan_SSB51829/m110218_185803_Spa_p4_b15.bas.h5) |
|  |  |  | **5** | [Pulse calling file](http://www.pacificbiosciences.com/devnet/files/datasets/publications/RNA_SmrtSeq/mRNA_data/1359/ivilfan_SSB51829/m110218_190442_Spa_p5_b15.pls.h5) ; [Base calling file](http://www.pacificbiosciences.com/devnet/files/datasets/publications/RNA_SmrtSeq/mRNA_data/1359/ivilfan_SSB51829/m110218_190442_Spa_p5_b15.bas.h5) |
|  |  |  | **6** | [Pulse calling file](http://www.pacificbiosciences.com/devnet/files/datasets/publications/RNA_SmrtSeq/mRNA_data/1359/ivilfan_SSB51829/m110218_192451_Spa_p6_b15.pls.h5) ; [Base calling file](http://www.pacificbiosciences.com/devnet/files/datasets/publications/RNA_SmrtSeq/mRNA_data/1359/ivilfan_SSB51829/m110218_192451_Spa_p6_b15.bas.h5) |
|  |  |  | **7** | [Pulse calling file](http://www.pacificbiosciences.com/devnet/files/datasets/publications/RNA_SmrtSeq/mRNA_data/1359/ivilfan_SSB51829/m110218_193143_Spa_p7_b15.pls.h5) ; [Base calling file](http://www.pacificbiosciences.com/devnet/files/datasets/publications/RNA_SmrtSeq/mRNA_data/1359/ivilfan_SSB51829/m110218_193143_Spa_p7_b15.bas.h5) |
|  |  |  | **8** | [Pulse calling file](http://www.pacificbiosciences.com/devnet/files/datasets/publications/RNA_SmrtSeq/mRNA_data/1359/ivilfan_SSB51829/m110218_193828_Spa_p8_b15.pls.h5) ; [Base calling file](http://www.pacificbiosciences.com/devnet/files/datasets/publications/RNA_SmrtSeq/mRNA_data/1359/ivilfan_SSB51829/m110218_193828_Spa_p8_b15.bas.h5) |
|  |  |  | **9** | [Pulse calling file](http://www.pacificbiosciences.com/devnet/files/datasets/publications/RNA_SmrtSeq/mRNA_data/1359/ivilfan_SSB51829/m110218_194529_Spa_p9_b15.pls.h5) ; [Base calling file](http://www.pacificbiosciences.com/devnet/files/datasets/publications/RNA_SmrtSeq/mRNA_data/1359/ivilfan_SSB51829/m110218_194529_Spa_p9_b15.bas.h5) |
|  |  |  | **10** | [Pulse calling file](http://www.pacificbiosciences.com/devnet/files/datasets/publications/RNA_SmrtSeq/mRNA_data/1359/ivilfan_SSB51829/m110218_195208_Spa_p10_b15.pls.h5) ; [Base calling file](http://www.pacificbiosciences.com/devnet/files/datasets/publications/RNA_SmrtSeq/mRNA_data/1359/ivilfan_SSB51829/m110218_195208_Spa_p10_b15.bas.h5) |
|  |  | **4** | **1** | [Pulse calling file](http://www.pacificbiosciences.com/devnet/files/datasets/publications/RNA_SmrtSeq/mRNA_data/1360/ivilfan_SSB51829/m110218_201552_Spa_p1_b15.pls.h5) ; [Base calling file](http://www.pacificbiosciences.com/devnet/files/datasets/publications/RNA_SmrtSeq/mRNA_data/1360/ivilfan_SSB51829/m110218_201552_Spa_p1_b15.bas.h5) |
|  |  |  | **2** | [Pulse calling file](http://www.pacificbiosciences.com/devnet/files/datasets/publications/RNA_SmrtSeq/mRNA_data/1360/ivilfan_SSB51829/m110218_202234_Spa_p2_b15.pls.h5) ; [Base calling file](http://www.pacificbiosciences.com/devnet/files/datasets/publications/RNA_SmrtSeq/mRNA_data/1360/ivilfan_SSB51829/m110218_202234_Spa_p2_b15.bas.h5) |
|  |  |  | **3** | [Pulse calling file](http://www.pacificbiosciences.com/devnet/files/datasets/publications/RNA_SmrtSeq/mRNA_data/1360/ivilfan_SSB51829/m110218_203718_Spa_p3_b15.pls.h5) ; [Base calling file](http://www.pacificbiosciences.com/devnet/files/datasets/publications/RNA_SmrtSeq/mRNA_data/1360/ivilfan_SSB51829/m110218_203718_Spa_p3_b15.bas.h5) |
|  |  |  | **4** | [Pulse calling file](http://www.pacificbiosciences.com/devnet/files/datasets/publications/RNA_SmrtSeq/mRNA_data/1360/ivilfan_SSB51829/m110218_204433_Spa_p4_b15.pls.h5) ; [Base calling file](http://www.pacificbiosciences.com/devnet/files/datasets/publications/RNA_SmrtSeq/mRNA_data/1360/ivilfan_SSB51829/m110218_204433_Spa_p4_b15.bas.h5) |
|  |  |  | **5** | [Pulse calling file](http://www.pacificbiosciences.com/devnet/files/datasets/publications/RNA_SmrtSeq/mRNA_data/1360/ivilfan_SSB51829/m110218_210029_Spa_p5_b15.pls.h5) ; [Base calling file](http://www.pacificbiosciences.com/devnet/files/datasets/publications/RNA_SmrtSeq/mRNA_data/1360/ivilfan_SSB51829/m110218_210029_Spa_p5_b15.bas.h5) |
|  |  |  | **6** | [Pulse calling file](http://www.pacificbiosciences.com/devnet/files/datasets/publications/RNA_SmrtSeq/mRNA_data/1360/ivilfan_SSB51829/m110218_211227_Spa_p6_b15.pls.h5) ; [Base calling file](http://www.pacificbiosciences.com/devnet/files/datasets/publications/RNA_SmrtSeq/mRNA_data/1360/ivilfan_SSB51829/m110218_211227_Spa_p6_b15.bas.h5) |
|  |  |  | **7** | [Pulse calling file](http://www.pacificbiosciences.com/devnet/files/datasets/publications/RNA_SmrtSeq/mRNA_data/1360/ivilfan_SSB51829/m110218_212754_Spa_p7_b15.pls.h5) ; [Base calling file](http://www.pacificbiosciences.com/devnet/files/datasets/publications/RNA_SmrtSeq/mRNA_data/1360/ivilfan_SSB51829/m110218_212754_Spa_p7_b15.bas.h5) |
|  |  |  | **8** | [Pulse calling file](http://www.pacificbiosciences.com/devnet/files/datasets/publications/RNA_SmrtSeq/mRNA_data/1360/ivilfan_SSB51829/m110218_213435_Spa_p8_b15.pls.h5) ; [Base calling file](http://www.pacificbiosciences.com/devnet/files/datasets/publications/RNA_SmrtSeq/mRNA_data/1360/ivilfan_SSB51829/m110218_213435_Spa_p8_b15.bas.h5) |
|  |  |  | **9** | [Pulse calling file](http://www.pacificbiosciences.com/devnet/files/datasets/publications/RNA_SmrtSeq/mRNA_data/1360/ivilfan_SSB51829/m110218_214939_Spa_p9_b15.pls.h5) ; [Base calling file](http://www.pacificbiosciences.com/devnet/files/datasets/publications/RNA_SmrtSeq/mRNA_data/1360/ivilfan_SSB51829/m110218_214939_Spa_p9_b15.bas.h5) |
|  |  |  | **10** | [Pulse calling file](http://www.pacificbiosciences.com/devnet/files/datasets/publications/RNA_SmrtSeq/mRNA_data/1360/ivilfan_SSB51829/m110218_215632_Spa_p10_b15.pls.h5) ; [Base calling file](http://www.pacificbiosciences.com/devnet/files/datasets/publications/RNA_SmrtSeq/mRNA_data/1360/ivilfan_SSB51829/m110218_215632_Spa_p10_b15.bas.h5) |

**Supplementary Table S5** A list links to the raw data. The links to the pulse and base calling data (fifth column) are provided for each sequencing run used to generate the data set published in this manuscript. The links are grouped according to the RNA template that was used in a particular experiment (first column). In addition, we state which Figure in the Main Manuscript and Supplementary Material includes each set of the data (second column). Each sequencing run (third column) typically included one movie (fourth column) except sequencing runs with *16S rRNA* and *mRNA* that include 10 movies per sequencing run. These were taken in a series with ZMW array alignment steps between individual movies (Methods).
